# Establishment of an *in vitro* RNA polymerase transcription system: a new tool to study transcriptional activation in *Borrelia burgdorferi*

**DOI:** 10.1038/s41598-020-65104-y

**Published:** 2020-05-19

**Authors:** William K. Boyle, Laura S. Hall, Anthony A. Armstrong, Daniel P. Dulebohn, D. Scott Samuels, Frank C. Gherardini, Travis J. Bourret

**Affiliations:** 1 Department of Medical Microbiology and Immunology, Creighton University, Omaha, NE 68105 USA; 2 Laboratory of Bacteriology, Gene Regulation Section, Division of Intramural Research, Rocky Mountain Laboratories, National Institute of Allergy and Infectious Diseases, National Institutes of Health, Hamilton, MT 59840 USA; 3 Division of Biological Sciences, University of Montana, Missoula, MT 59812 USA; 4 Bioinformatics and Computational Biosciences Branch, Office of Cyber Infrastructure and Computational Biology, National Institute of Allergy and Infectious Diseases, National Institutes of Health, Bethesda, MD 20892 USA

**Keywords:** Holoenzymes, Bacterial transcription

## Abstract

The Lyme disease spirochete *Borrelia burgdorferi* exhibits dramatic changes in gene expression as it transits between its tick vector and vertebrate host. A major hurdle to understanding the mechanisms underlying gene regulation in *B. burgdorferi* has been the lack of a functional assay to test how gene regulatory proteins and sigma factors interact with RNA polymerase to direct transcription. To gain mechanistic insight into transcriptional control in *B. burgdorferi*, and address sigma factor function and specificity, we developed an *in vitro* transcription assay using the *B. burgdorferi* RNA polymerase holoenzyme. We established reaction conditions for maximal RNA polymerase activity by optimizing pH, temperature, and the requirement for divalent metals. Using this assay system, we analyzed the promoter specificity of the housekeeping sigma factor RpoD to promoters encoding previously identified RpoD consensus sequences in *B. burgdorferi*. Collectively, this study established an *in vitro* transcription assay that revealed RpoD-dependent promoter selectivity by RNA polymerase and the requirement of specific metal cofactors for maximal RNA polymerase activity. The establishment of this functional assay will facilitate molecular and biochemical studies on how gene regulatory proteins and sigma factors exert control of gene expression in *B. burgdorferi* required for the completion of its enzootic cycle.

## Introduction

*Borrelia burgdorferi* is a highly fastidious host-associated bacterium in the phylum Spirochaetes^1,2^. *B. burgdorferi* is adapted to a vector-host life cycle and possesses a condensed 1.3 Mb genome with limited metabolic capability^3,4^. The genome lacks genes encoding components of the citric acid cycle, the electron transport chain, amino acid biosynthesis, and fatty acid biosynthesis pathways, wholly relying on the transport of sugars, fatty acids and amino acids from the environment for survival^5,6^. *B. burgdorferi* growth occurs extracellularly in vertebrate tissues and in ticks following a blood meal, which poses additional nutritional constraints^7^. In particular, the host competition for iron is hypothesized to have resulted in abstinence from iron utilization by *B. burgdorferi*^8–10^. Instead, manganese is thought to largely replace iron as a metal cofactor for metabolic enzymes^9,11^.

*B. burgdorferi* regulates gene expression to adapt to environmental constraints faced in its enzootic cycle. *B. burgdorferi* responds to environmental changes in pH, temperature, nutrient availability, and manganese levels with dramatic shifts in transcription and growth^12–18^. The transcriptional changes in *B. burgdorferi* during transmission from the arthropod vector *Ixodes scapularis*^19,20^ are thought to be influenced by the mechanisms underlying environmental sensing and transcriptional responses. Mechanistic studies of how transcription factors regulate gene expression in *B. burgdorferi* have been hindered by the scarcity of biochemical tools. To understand the transcriptional mechanisms that support *B. burgdorferi* survival, we set out to characterize the RNA polymerase of *B. burgdorferi*.

To develop a *B. burgdorferi* specific *in vitro* transcription assay, we purified intact RNA polymerase core enzyme, and the housekeeping sigma factor RpoD (σ^70^). Bacterial RNA polymerases are composed of four polypeptide subunits designated α, β, β′, and σ. The largest subunit β′ houses the active site that catalyzes RNA synthesis. In the cell, the β′ subunit is associated with the β and α subunits and forms the core enzyme composed of the two α subunits, one β subunit, and one β′ subunit that is capable of transcriptional elongation on single-stranded DNA^21^. The σ subunit is required for the recognition of the promoter and opening of the DNA double helix^22^. An additional, small subunit, ω, is also known to promote polymerase activity^23^. In this study, we characterized the subunit composition, reaction conditions, and sigma factor-dependent transcriptional activity of RNA polymerase purified from *B. burgdorferi*. We assayed previously annotated *B. burgdorferi* transcriptional start sites using RpoD to demonstrate the utility of this new *in vitro* transcription system.

## Results

### Structural model of the *B. burgdorferi* RNA polymerase

**Figure 1:**
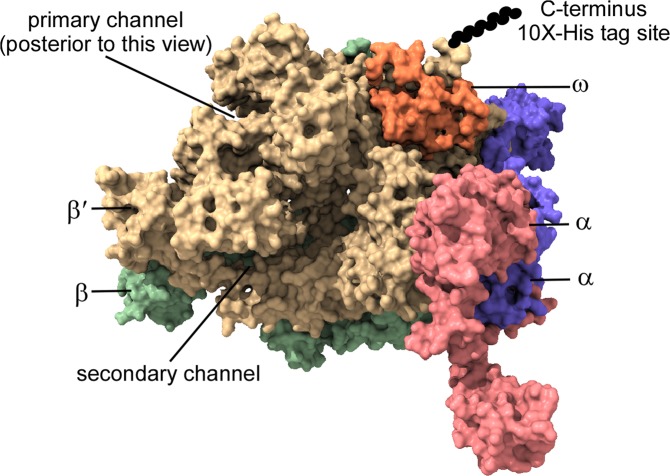
Model of the *B. burgdorferi* RNA polymerase core. The *B. burgdorferi* RNA polymerase core model was created by modeling the subunits β′ (yellow), β (green), α (pink and purple), and ω (orange) individually using Iterative Threading Assembly Refinement (I-TASSER) and subsequently aligning the modeled subunits to the *E. coli* RNA polymerase (PDB 3lu0) in PyMOL. Location of the affinity tag appended to C-terminus of the β′ subunit is shown schematically as a chain of black spheres and labeled.

A model of the *B. burgdorferi* RNA polymerase core enzyme was generated to evaluate its structure for affinity chromatography purification. Individual models of β′, β, and α, subunits were generated with I-TASSER^24^ and aligned to a model of *E. coli* RNA polymerase (3lu0)^25^. An additional subunit, ω, which complexes with the RNA polymerase core at the carboxyl terminus of the β′ subunit, was included in the model to assess its influence on structure. Only minimal relaxation of the aligned model was necessary to prevent clash at the inter-protomer interfaces. Purification of an active RNA polymerase core from other bacteria has been achieved through affinity chromatography strategies that result in co-purification of the RNA polymerase protein complex^26^. Typically, the β′ subunit is appended with a carboxyl-terminal affinity-tag. The potential location of the β′ subunit carboxy-terminus is illustrated in Fig. 1. This model revealed that the carboxyl terminus of the β′ subunit is surface exposed and away from the interface between the β′ subunit and other subunits. We generated a *B. burgdorferi* strain harboring a sequence encoding a polyhistidine-tag on the 3′ end of *rpoC* (encoding the RNA polymerase β′ subunit) by homologous recombination and designated the strain 5A4-RpoC-His10X.

#### Purification of RNA polymerase

**Figure 2:**
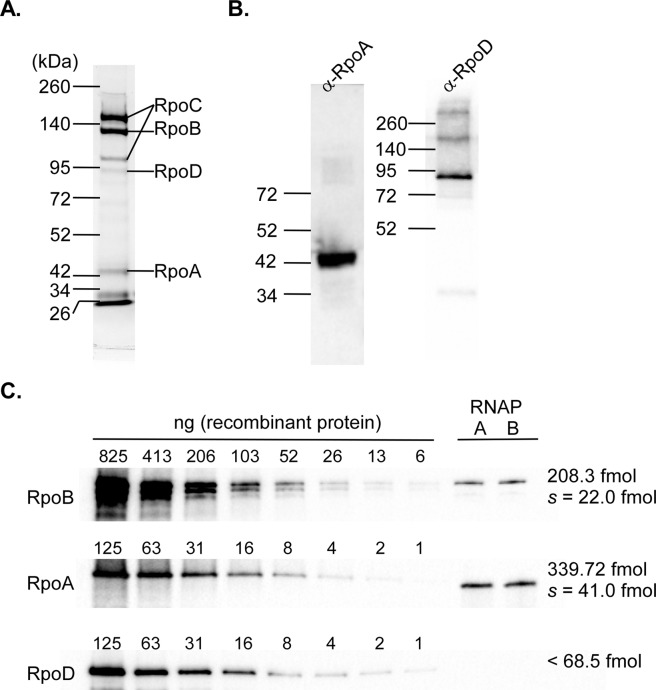
Purification of the RNA polymerase from *B. burgdorferi* and determination of the molar ratio of the core subunits β and α. (**A**) Purified proteins in pooled elution fraction from nickel-affinity chromatography performed on lysates generated from *B. burgdorferi* 5A4-RpoC-His10X were separated by SDS-PAGE and stained with Coomassie Brilliant Blue. Labels on the right side of the gel indicate RNA polymerase subunits detected by LC-MS of excised bands. (**B**) Western blots were performed on nickel-affinity purified proteins using anti-*Borrelia*-RpoA and anti-*Borrelia*-RpoD antibodies to confirm the presence of the target proteins. Numbers indicate the migration of protein molecular mass markers. Detection of RpoD required loading microgram quantities of purified RNA polymerase. (**C**) Molar ratios were determined by quantitative western blots. Recombinant RpoB, RpoA, and RpoD were loaded in amounts indicated above to form a standard curve. Purified RNA polymerase samples A and B were loaded with the standard curve for quantification. Molar amounts were calculated from the theoretical masses of proteins based on amino acid sequence. Images are representative of four replicate experiments.

To test if the *B. burgdorferi* RNA polymerase would co-purify with the affinity-tagged β′ subunit, 3–4 L cultures of 5A4-RpoC-His10X were grown to mid-logarithmic growth phase (~5 × 10^7^ spirochetes · ml^−1^), and cell lysates generated using a French pressure cell were subjected to nickel-metal affinity chromatography. The presence of RNA polymerase in the elution fractions was analyzed by SDS-PAGE and staining of proteins with Coomassie Brilliant Blue. Elution fractions containing RNA polymerase were pooled and yielded 40–50 µg of extracted protein. The components of the RNA polymerase holoenzyme (β′, β, α, and σ) in the pooled elution fractions were identified by LC-MS following separation of proteins by SDS-PAGE and in-gel digestion (Fig. 2A). LC-MS identified the most heavily Coomassie-stained gel bands as subunits β′, β, and α migrating at their expected protein sizes (154, 129, and 38.5 kDa, respectively). Among proteins identified in the elution fraction mixture were likely RpoC and RpoB cleavage products (one of which is indicated on Fig. 2A). Some of the unquantified contaminating proteins likely interact with RNA polymerase, including RNA polymerase secondary channel binding protein GreA, transcription termination/antitermination protein NusA, chaperone protein DnaK, as well as several ribosomal proteins. Gel images and results from LC-MS are available in Fig. S1 and Table S1. Notably, proteins closely associated with the RNA polymerase complex such as the ω subunit and alternative sigma factors RpoS and RpoN were not identified by LC-MS. Previous studies suggest alternative sigma factors are not expressed at high levels during logarithmic growth in culture^27–29^. Consistent with these previous studies, alternative sigma factors did not appear to co-purify with RNA polymerase isolated from logarithmic phase *B. burgdorferi* cultures. The presence and migration of the co-purified RNA polymerase subunits α and σ^70^ were subsequently confirmed by western blot analysis using polyclonal antibodies raised against recombinant *B. burgdorferi* RpoA and RpoD (Fig. 2B). Together, these results indicate *B. burgdorferi* RNA polymerase holoenzyme subunits co-purify under the affinity chromatography conditions tested allowing for affinity purified RNA polymerase enzymatic activity to be examined.

### Initial characterization of the RNA polymerase from *B. burgdorferi*

To accurately measure the relative amounts of the subunits from purified RNA polymerase, the concentration of the β, α, and σ subunits were measured by quantitative western blot analyses. Linear detection ranges were determined for the western blots developed with *B. burgdorferi* RNA polymerase subunit-specific polyclonal antibodies anti-RpoB, anti-RpoA, and anti-RpoD by loading known quantities of the respective purified recombinant RpoB, RpoA, and RpoD proteins (Fig. 2C). Chemiluminescent signals resulting from the western blots were analyzed by densitometry to determine the linear range of detection for each protein. Quantitative western blots performed with 100 ng of affinity purified RNA polymerase produced chemiluminescence signals within the linear range of detection for assays using anti-RpoB and anti-RpoA. There was an insufficient amount of sigma factor within 100 ng of the affinity purified RNA polymerase mixture to yield a signal within the linear range of detection with anti-RpoD, indicating fewer sigma factors were co-purified through affinity chromatography (˂68.5 fmol/100 ng). Given a measured concentration of 340 fmol of α subunit and 208 fmol of β subunit per 100 ng, an estimate of the molar ratio from the affinity purified RNA polymerase was 1.63:1 (α:β subunits). Canonically, an active RNA polymerase core contains a minimum of four subunits (β′, β, two α), and a 2:1 ratio of α subunit to β subunit is expected. Consequently, we reasoned that the maximum concentration of fully constituted RNA polymerase in our affinity purified RNA polymerase sample was 170 fmols per 100 ng of protein with α as the limiting subunit. The molar concentrations of RNA polymerase indicated for all subsequent experiments described here are expressed as the values determined by quantitative western blot analyses.

To determine reaction conditions that support RNA polymerase activity, we utilized a dye-incorporation method for detection of RNA synthesis that allowed for various reaction conditions to be screened^30,31^. Circular, single-stranded DNA was used as a template because it relieved the requirement for sigma factor-dependent transcriptional initiation as RNA polymerase requires a sigma factor to initiate transcription from dsDNA. A circular, single-stranded DNA template also allows for the accumulation of long RNA transcripts that are easily detectable using intercalating dyes. Circular, single-stranded DNA ranging in size from 45 to 180 nucleotides were generated as templates for the RNA polymerase. RNA polymerase activity was tested under various reaction conditions by adding potassium glutamate, magnesium chloride (MgCl_2_), zinc chloride, calcium chloride, and/or manganese chloride (MnCl_2_) to a base buffer containing a final concentration of 40 mM HEPES, pH 7.5, 0.05% NP40, and 1 mM DTT. Initial *in vitro* transcription reaction mixtures containing 100 nM DNA template, 100 nM RNA polymerase, and 200 µM nucleotides (NTPs) were incubated for 5–6 hours at 37 °C to allow for the accumulation of RNA transcripts (Fig. S2). Our data indicated reactions containing 12 mM MgCl_2_ or 2–10 mM MnCl_2_ had detectable levels of RNA product accumulation based on SYBR Safe dye incorporation suggesting a metal requirement for RNA polymerase enzymatic activity. Further characterization determined RNA products were detectable within minutes when RNA polymerase was pre-incubated with 2–10 mM MnCl_2_ prior to addition of the DNA template to initiate the reaction. Collectively, these experiments established initial reaction conditions for achieving enzymatic activity from affinity-purified RNA polymerase. Subsequent reactions to test RNA polymerase activity from dsDNA templates were carried out in 60 mM potassium glutamate, 2 mM MgCl_2_, and 5 mM MnCl_2_ in addition to the base buffer described above.

### RNA polymerase activity from double-stranded DNA templates

**Figure 3:**
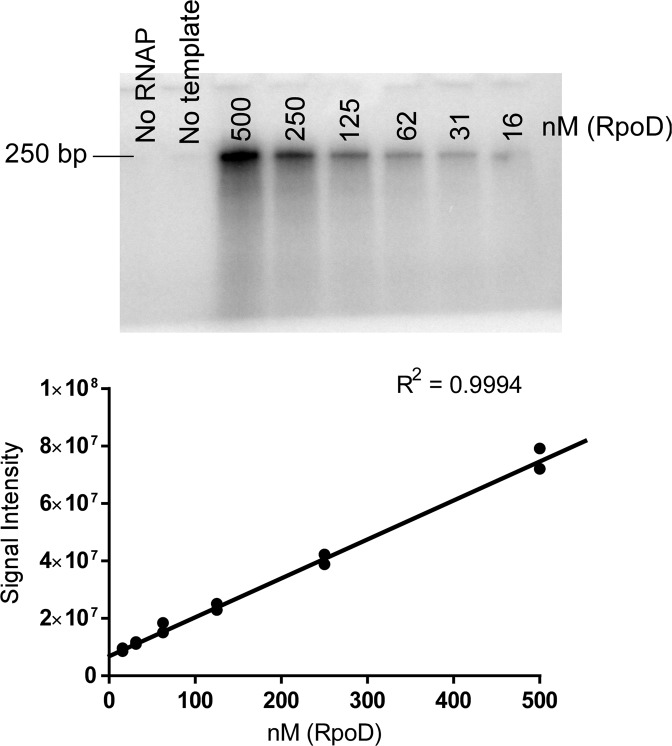
The accumulation of RNA products from *in vitro* transcription reactions using *flgB* promoter dsDNA increases with increasing concentrations of the sigma factor RpoD. RNA was separated by a 10% TBE-urea gel and radiolabeled RNA was detected by phosphor screen. The signal from the phosphor screen was quantified by densitometry. Results are representative of two replicate experiments. R^2^ for linear regression is reported to indicate RpoD-dependent variability in RNA polymerase activity.

We next generated a linear dsDNA template by amplifying the *flgB* promoter (*flgBp*) from *B. burgdorferi* genomic DNA by PCR. The *flgB* promoter is RpoD-dependent^32^ and the 499-bp PCR product encompasses a region from −248 to +251 surrounding the transcription initiation site. Levels of RpoD required for RNA polymerase activity initiated from the *flgB* promoter were determined by titration of recombinant RpoD in the reactions (Fig. 3). Reaction mixtures containing 21 nM RNA polymerase and 10 nM of the linear dsDNA template encoding the *flgB* promoter were supplemented with 16–500 nM of recombinant RpoD and the accumulation of RNA products was quantified by the incorporation of α-^32^P-ATP. The accumulation of RNA products in the reaction increased linearly with increasing concentrations of RpoD. To maximize the rate of transcriptional initiation, subsequent *in vitro* transcription reactions were carried out with 500 nM RpoD in the reaction mixture at a 24:1 molar ratio of RpoD to RNA polymerase.

### RNA polymerase activity is pH- and temperature-dependent

**Figure 4:**
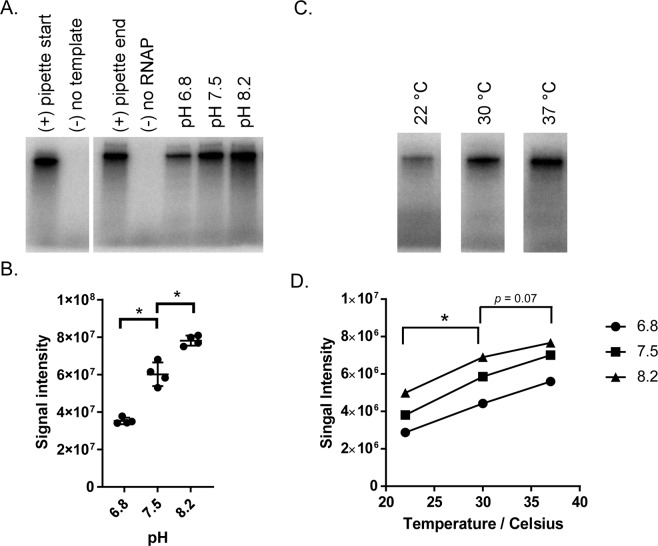
pH and temperature ranges for RNA polymerase activity. (**A)** RNA products from *in vitro* transcription reactions conducted at 30 °C and pH 6.8, 7.5, or 8.2 were separated on 10% TBE-urea gel. Radiolabeled RNA was detected by phosphor imaging. Reactions were initiated individually, and controls were added for transcription initiation timing (pipette start and end). (**B**) Signal intensity on the phosphor screen was determined by densitometry using data collected from four replicate experiments. Asterisks indicate *p*-value of <0.001 in a comparison of signal intensity between pH values using a one-way ANOVA. (**C**) Representative image of RNA products generated from *in vitro* transcription reactions carried out at 22, 30, or 37 °C, at pH 6.8 and separated on 10% TBE-urea gel. Cropped gels were generated from a single image. (**D**) pH and temperature conditions yielding the highest RNA polymerase activity. Signal intensities from phosphor imaging were determined by densitometry. Each point represents the average of two replicate experiments. Asterisks indicate *p*-value of <0.001 in a comparison of signal intensity achieved at different temperatures in a mixed-effects ANOVA to account for pH-based differences.

Having established reaction conditions that permit transcription from a linear dsDNA template with the *flgB* promoter, we assayed the activity of the RNA polymerase within a range of temperature and pH (Fig. 4). Utilizing a reaction containing the RNA polymerase, recombinant RpoD, and the *flgBp* template, three pH values encompassing the buffering range of HEPES (pH 6.8, 7.5, and 8.2) were tested at 30 °C (Fig. 4A,B). The accumulation of the RNA products was detected by the incorporation of α-^32^P-ATP. Accumulation of RNA products increased with increasing pH; RNA polymerase had the highest transcriptional activity at pH 8.2. Additional reactions using the three pH values and three temperature conditions, 22 °C, 30 °C, and 37 °C, within the range encountered by *B. burgdorferi* during its enzootic cycle, were performed (Fig. 4C,D). Accumulation of RNA products increased with increased temperature; RNA polymerase activity was highest at 37 °C. These results indicate RNA polymerase activity responds to temperature and pH, which is consistent with previously characterized bacterial RNA polymerases^33–35^. In addition, we observed reactions performed at pH 8.2 do not permit use of pre-mixed reaction buffers, likely due to the instability of DTT at high pH, which significantly reduces its half-life^36^. Therefore, subsequent *in vitro* transcription reactions were carried out at pH 7.5 and 37 °C unless indicated otherwise. These RNA polymerase reaction conditions yielded the highest and most reproducible activity in this study.

### *B. burgdorferi* RNA polymerase requires manganese for activity

**Figure 5:**
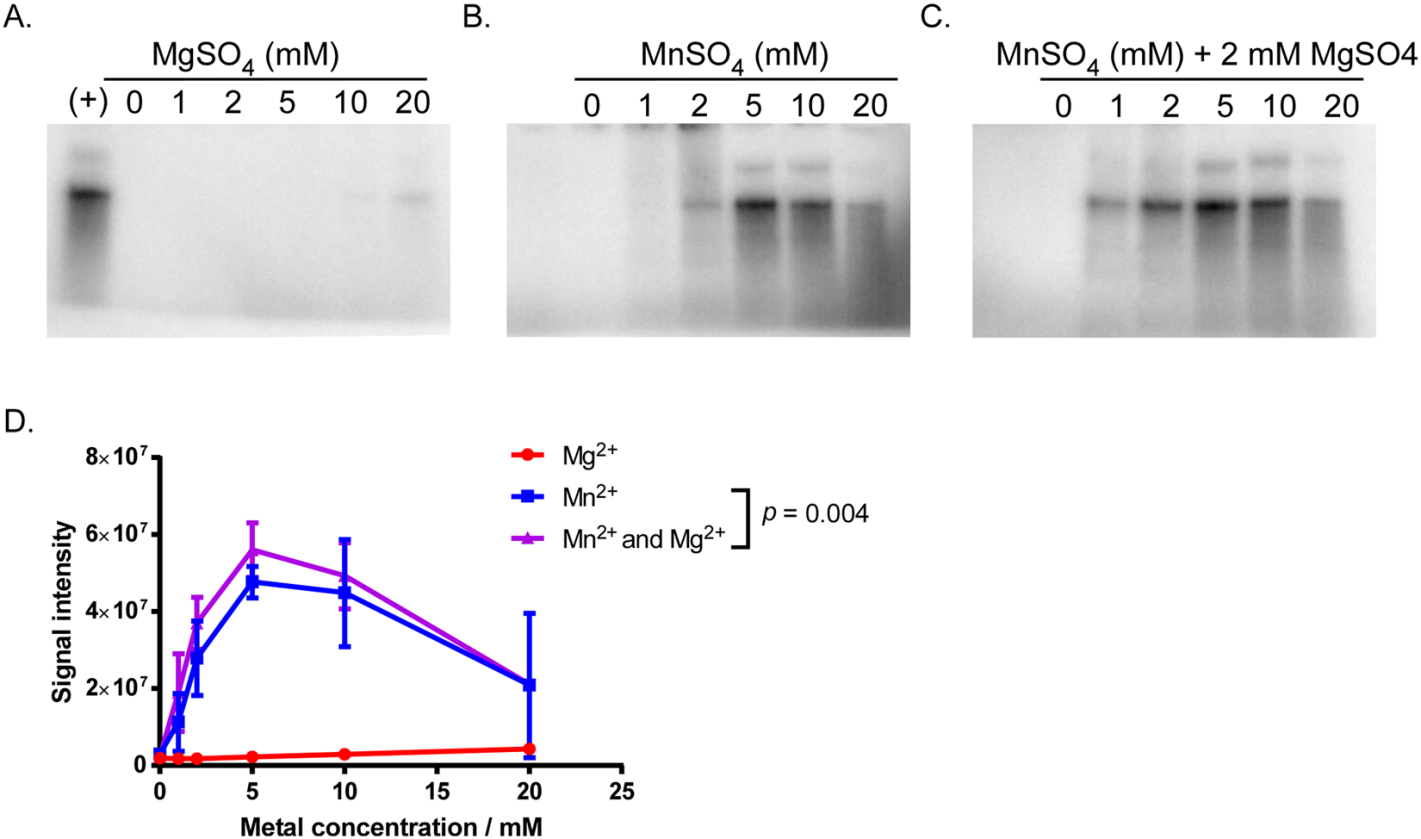
Evaluation of magnesium and manganese requirement on RNA polymerase activity. RNA products generated from *in vitro* transcription assays containing various concentrations of magnesium and manganese metal ions were separated by gel electrophoresis in a 10% TBE-Urea gel and detected using a phosphor screen. Representative gels are shown from *in vitro* transcription reactions prepared in parallel with metal salts: reactions containing (**A**) 0–20 mM magnesium or (**B**) 0–20 mM manganese or (**C**) 2 mM magnesium with 0–20 mM manganese. Data are representative of three replicate experiments and gels were cropped from a single image. Phosphor screen signals were quantified by densitometry and plotted over varied magnesium or manganese concentrations (**D**). Differences in signal intensity achieved using manganese alone (1–20 mM Mn^2+^) and those supplemented with magnesium (1–20 mM Mn^2+^ with 2 mM Mg^2+^) were analyzed by a two tailed paired *t*-test matched along the entire metal concentration range and was found to be statistically significant (α = 0.05, *p* = 0.004), indicating a role for magnesium in RNAP activity.

Initial screening for RNA polymerase activity indicated that magnesium or manganese is required for activity using single-stranded DNA templates (Fig. S2). Therefore, we screened the metal-dependent activity of RNA polymerase holoenzyme with recombinant RpoD, using the dsDNA *flgBp* template (Fig. 5). We tested the magnesium- and manganese-dependent activity of the RNA polymerase using ultra high purity (>99.9%) metal salts containing sulfate anions to remove potential noise from trace contamination. We observed a lower concentration of manganese, compared to magnesium, is required for activity. Reaction buffers containing 0–20 mM magnesium sulfate (MgSO_4_), 0–20 mM manganese sulfate (MnSO_4_), or 2 mM MgSO_4_ with 0–20 mM MnSO_4_ were prepared in parallel (Fig. 5A–C). The accumulation of RNA products over 5 min was measured following the addition of the dsDNA template. *In vitro* transcription reactions containing MgSO_4_ required a 20 mM concentration for detectable RNA polymerase activity, while reaction mixtures containing MnSO_4_ required tenfold less (2 mM) manganese for detectable activity and did not require magnesium ions in the reaction mixture. Reaction mixtures containing 2 mM MgSO_4_ along with varying levels of MnSO_4_ all had higher levels of activity compared to reaction mixtures with only MnSO_4_ (Fig. 5D). These results indicate manganese is required for *B. burgdorferi* RNA polymerase activity whereas magnesium plays a supplementary role.

**Figure 6:**
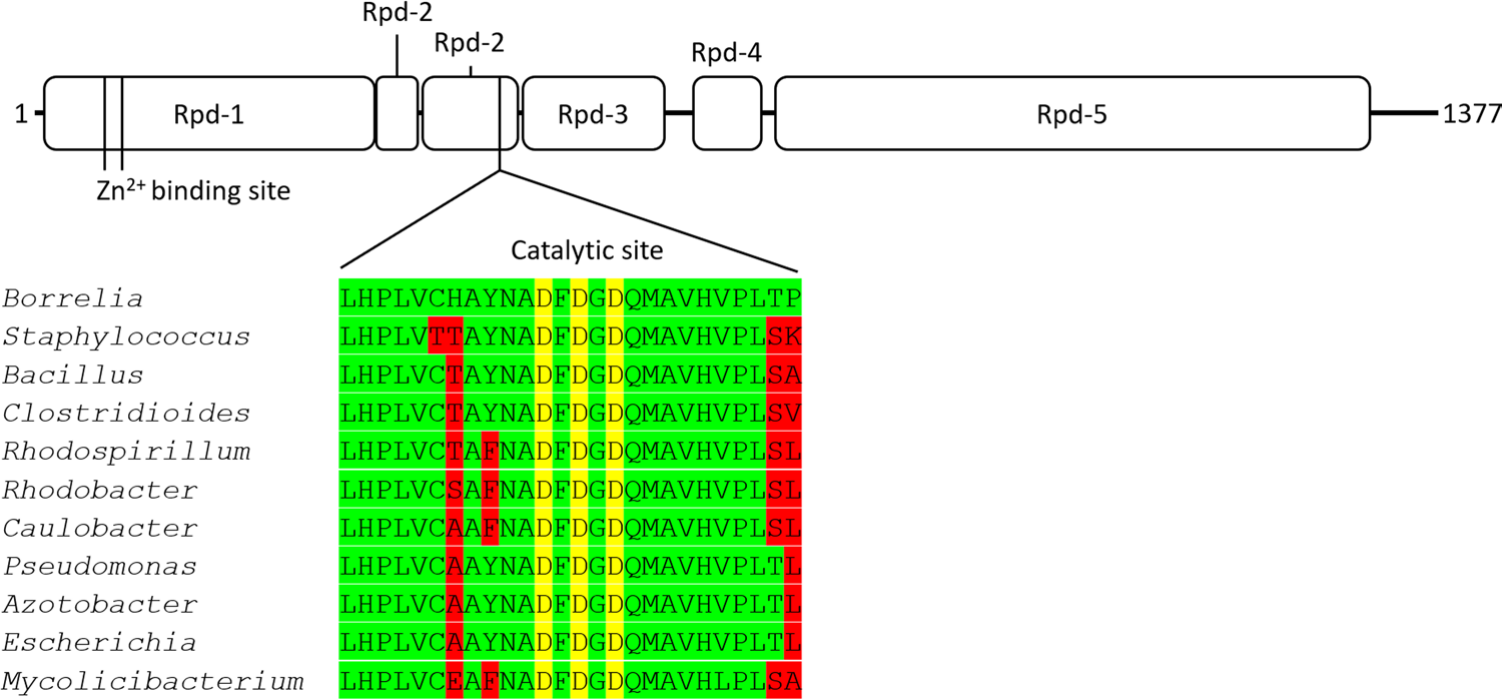
RNA polymerase domains (Rpd) and metal binding sites of *B. burgdorferi* RpoC and alignment of the amino acids at the catalytic site with other bacterial species. Genus names from which the RNA polymerases were purified and characterized are shown alongside the amino acid alignment of the catalytic site. Aspartates in the positions highlighted in yellow coordinate catalytic metal ions, typically magnesium. Amino acid positions in red indicate positions where the amino acid identity differs from *Borrelia*.

The amino acid sequence of *B. burgdorferi* RNA polymerase β′ subunit was aligned to β′ subunits from other bacterial species to better understand the role of manganese using Clustal Omega and MSAProbs amino acid sequence alignment algorithms utilizing hidden Markov models^37,38^. Gram positive species in the genera *Bacillus* and *Clostridium* possess RNA polymerases that are enhanced by the presence of manganese^39,40^. The β′ subunits of these bacteria were included in the alignment to the β′ subunit in *B. burgdorferi*, along with prototypical magnesium-dependent RNA polymerases from other genera. However, the alignment around the active site, which binds magnesium, revealed no conserved pattern among the manganese-associating RNA polymerases (Fig. 6). A full alignment of β′ subunit amino acid sequences are available in Table S3. Therefore, which divalent cation incorporates into the active site of the *B. burgdorferi* RNA polymerase remains unclear.

### RpoD-dependent promoter selectivity

**Figure 7:**
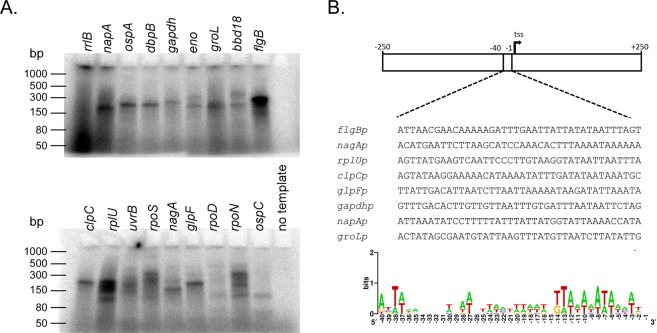
Promoter selection by the RNAP holoenzyme. *In vitro* transcription reactions were initiated with dsDNA templates encompassing the predicted promoters upstream of the indicated genes. The genes are ordered from highest to lowest number of transcripts during *in vitro* culture at the transcription initiation site as measured by RNA-seq reported in a previously published data set^41^. (**A**) The RNA products were separated by gel electrophoresis and detected by phosphor imaging. Gels were cropped from a single image and are representative of three independent experiments. (**B**) A consensus sequence was generated from DNA sequence encoded in −40 to −1 position from the transcription start sites using sequence logo. Seven templates that encode promoters to housekeeping genes and produce single RNA products were chosen to generate the sequence.

We next tested if the RNA polymerase holoenzyme can select various promoter sites encoded within the *B. burgdorferi* genome. Defined transcriptional initiation sites were chosen from an RNA-seq data set that mapped processed and unprocessed RNA 5′ ends to define transcription start sites^41^. dsDNA templates for 18 *B. burgdorferi* genes were generated by PCR encompassing the predicted promoter regions. DNA template size, relative promoter strength, and transcript size based on known transcriptional start sites are found in Table 1. *In vitro* transcription reactions were carried out using the 18 dsDNA templates. Reaction products were separated by gel electrophoresis to detect the relative product size and quantity (Fig. 7A). Reactions using each of the dsDNA templates generated products matching the expected transcript sizes (Table 1). A consensus sequence for the RpoD-dependent promoter site was generated using seven templates encoding promoters to housekeeping genes that yielded the strongest relative signals (*flgBp*, *nagAp*, *rplUp, clpCp, glpFp, gapdhp, napAp*, and *groLp*) using previously annotated transcription initiation sites (Fig. 7B). Conserved sequences in position −1 to −40 determined by sequence logo resemble AT-rich promoter sequence previously determined by the MEME motif discovery algorithm^41,42^. While multiple time points or a promoter competition experiment would be required to properly quantify relative signal strengths from the various templates, the strongest signals were repeatedly generated from *flgBp* and *rplUp*, and signals generated from *ospCp* and *bbd18p* were among the weakest. These observations were consistent with previous studies showing *flgBp* is a strong RpoD-dependent promoter and *ospCp* is an RpoS-dependent promoter^29,32,43^. Together, these results suggest the RpoD-directed RNA polymerase holoenzyme preferentially selects certain promoters (*flgBp* and *rplUp*) in our *in vitro* transcription system.

**Table 1:** Oligonucleotide size for each promoter template.

Template	Annotated promoter location	Annotated relative expression signal	Template size (bp)	Predicted transcript size
*rrlBp*	chr:438576.0.438575 (−strand)	23421	526	307
*napAp*	chr:731171.0.731170 (+ strand)	8164	486	200
*ospAp*	lp54:9422.0.9421 (+ strand)	8119	498	202
*dbpBp*	lp54:17935.0.17934 (−strand)	7871	492	204
*gapdhp*	chr:53495.0.53494 (−strand)	4175	483	224
*enop*	chr:345027.0.345026 (+ strand)	3164	500	194
*groLp*	chr:688446.0.688445 (+ strand)	2852	498	199
*bbd18p*	lp17:11692.0.11691 (−strand)	2612	491	198
*flgBp*	chr:303772.0.303771 (−strand)	2540	499	254
*clpCp*	chr:886498.0.886497 (−strand)	1553	543	228
*rplUp*	chr:818553.0.818552 (+ strand)	1263	484	239
*uvrBp*	chr:888243.0.888242 (+ strand)	1182	504	200
*rpoSp*	chr:813934.0.813933 (−strand)	752	534	224 (long) 112 (short)
*nagAp*	chr:150912.0.150911 (+ strand)	658	470	182
*glpFp*	chr:245596.0.245595 (+ strand)	565	511	217
*rpoDp*	chr:747681.0.747680 (+ strand)	266	484	190
*rpoNp*	chr:472376.0.472375 (−strand)	64	509	318
*ospCp*	cp26:16884.0.16883 (+ strand)	38	508	211

## Discussion

In this study, an intact RNA polymerase complex was purified from *B. burgdorferi* by affinity chromatography, and an *in vitro* transcription assay was developed by establishing buffer and metal concentrations for reaction mixtures. The activity of RNA polymerase in a range of pH and temperature was demonstrated. We leveraged this *in vitro* transcription system to assay RpoD promoter selection on previously annotated promoters. These promoter sites were recognized by RpoD, as evidenced by specific length products produced by the RNA polymerase. This demonstrates the utility of these transcriptional start sites previously assayed and annotated in a 5′ end transcriptome and makes possible an assay of RpoD-dependent transcriptional initiation.

The level of RNA polymerase activity we detected from various promoters differed qualitatively in signal strength. The qualitative differences in promoter strength observed in our *in vitro* transcription assays does not necessarily closely correlate with relative strength of transcriptional start sites measured from live cells. For example, *ospAp*, *clpCp*, and *glpFp* all produce *in vitro* transcription products of similar signal intensity (Fig. 7), although, the relative transcriptional starts from these promoters differ by two orders of magnitude in cells (Table 1). The interactions of many transcription factors with bacterial RNA polymerase determine the rates of transcriptional initiation *in vivo*. Transcription initiation from a promoter site may vary not only by sigma factor binding, but also by accessibility of the site due to steric hindrance, polymerase availability, or DNA supercoiling^44^. Moreover, there was no promoter competition in the *in vitro* transcription reactions to compare relative promoter strength, as has been engineered in other systems^45^. This limits the interpretation of relative signal strength produced in the *in vitro* transcription reactions described in this study. For example, *dbpBp*, which is thought to be primarily recognized by RpoS, is also recognized by RpoD, but the relative signals obtained from our assays should not be used to infer promoter strength^29^. Further investigations that include the use of alternative sigma factors and transcription factors will be required to resolve the apparent discrepancies between what we observed in the *in vitro* transcription assays presented here and what occurs in live cells.

Major components in the nickel affinity purified RNA polymerase mixture were identified as the homologs of β′, β, and α subunits by immunoblotting and LC-MS. No other subunits were required for RNA polymerase transcriptional activity using single-stranded DNA templates. Quantitative western blotting determined that the α subunit was the most abundant subunit in the mixture but constituted less than twofold the number of β′ and β subunits, suggesting *B. burgdorferi* RNA polymerase enzyme consists of the typical β′βα_2_ arrangement with only a portion of the RNA polymerases carrying both α subunits. We suspect some of the α subunits are capable of dissociating either in the cell or during the purification process to produce non-productive RNA polymerases in the mixture, thereby limiting maximal enzymatic activity. Similarly, a significantly smaller quantity of RpoD was co-purified with the RNA polymerase, requiring supplementation with additional sigma factor subunits for measurable RNA polymerase activity from dsDNA templates. Similarly, the ω subunit, considered part of the RNA polymerase complex, did not co-purify with the core. Previous reports suggest the interaction of ω subunit with the RNA polymerase can be transient, therefore, this subunit may have been lost during purification^46^. Further investigations will be required to characterize the role of the ω subunit on *B. burgdorferi* RNA polymerase activity.

Other co-purified products present in the mixture were not in apparent abundance when observing the Coomassie-stained gel and included proteases and transcription factors (Table S1). While bands excised from the stained electrophoresis gels of the purified RNA polymerase contained peptides other than subunits of RNA polymerase core, these results are not surprising. Other RNA polymerase complexes purified using strategies similar to those presented here contain detectable contaminants^47–50^. Our study differed from these previous purification efforts only because we identified the contaminating peptides by highly sensitive LC-MS. Nevertheless, this study did not rule out a role for these factors that may interact with subunits of the RNA polymerase and alter its activity. Undoubtedly, a higher purity RNA polymerase will be required for structural studies; however, the level of RNA polymerase purity shown in this study was sufficient to observe and quantify activity from specific *B. burgdorferi* promoters.

In this study, we determined that RNA polymerase of *B. burgdorferi* is likely dependent on manganese. Manganese is a common contaminant in MgCl_2_ stocks and utilizing ultrapure magnesium revealed the requirement for manganese for *B. burgdorferi* RNA polymerase activity. Manganese was required for transcription elongation from both ssDNA and dsDNA templates *in vitro*. These observations suggest that manganese in *B. burgdorferi* RNA polymerase is not required for association with dsDNA or with a sigma factor (when RNA polymerase interacts with ssDNA). While prototypical bacterial RNA polymerases are thought to be magnesium-dependent^51^, RNA polymerases purified from the Firmicutes (*Clostridium acetobutylicum, Bacillus subtilis*, and *Lactobacillus curvatus*) are manganese-dependent^40,52,53^. A regulatory role for manganese has been demonstrated for some previously characterized RNA polymerases, such as transcription stalling, while others seem to require manganese for catalytic activity^40,51–56^. Although the highest level of activity from the *B. burgdorferi* RNA polymerase was obtained from a combination of magnesium and manganese, the role of manganese in catalysis remains unexplored; note that the conserved catalytic site amino acid residues do not differ between magnesium- and manganese-utilizing bacteria. Moreover, the possibility that metals such as magnesium may co-purify with the RNA polymerase was not ruled out. Further structural characterization is required to determine the exact roles of manganese and magnesium for *B. burgdorferi* RNA polymerase activity.

A unique feature of *B. burgdorferi* physiology is its accumulation of manganese in the intracellular environment along with the apparent lack of intracellular iron^9,10^. The role of manganese in the *B. burgdorferi* RNA polymerase should be carefully considered due to its numerous potential roles in physiology. Manganese levels in *B. burgdorferi* are maintained by the BmtA transporter^57,58^, and spirochetes actively accumulate manganese in the cytosol. Levels of intracellular manganese in *B. burgdorferi* can change depending on the manganese concentration in the culture media^14,57^. Additionally, manganese levels were shown to affect the transcription of virulence genes and are hypothesized to be an environmental cue required for *B. burgdorferi* transmission^14,59^. The impact of varying concentrations of manganese on RNA polymerase activity in this study suggests regulation of optimal intracellular levels of manganese in *B. burgdorferi* are required to support basal transcription.

Currently, several transcription factors are proposed to respond to environmental cues and to regulate transcription initiation in *B. burgdorferi*. These transcription factors include Rrp2, BosR (the ferric uptake regulator homolog), BadR (the *Borrelia* host adaptation regulator), and DksA^13,60–66^. Our newly developed *in vitro* transcription assay can be used to confirm the function of these factors as activators or repressors of transcription from cognate promoters, which was not previously feasible for *B. burgdorferi*. Additionally, we observed RpoD only weakly drove transcriptional initiation from the *ospC* promoter, which is known to be regulated by the alternative sigma factor RpoS^29,67,68^. The data presented here demonstrate the utility of *in vitro* transcription assays to test the hypothesis that alternative sigma factors control transcription of dissimilar sets of genes in *B. burgdorferi*.

### Experimental procedures

#### RNA polymerase structure modeling

Amino acid sequences of the RNA polymerase subunits (NP_212636, YP_008686574, NP_212522.1, and NP_212954) were individually submitted to the Iterative Threading Assembly Refinement (I-TASSER) server for modeling with no template specification^69^. An alignment of the resulting I-TASSER models to the corresponding subunits of a molecular model of the *E. coli* RNA polymerase core deposited in the Protein Databank under accession number 3lu0^25^ was then performed with PyMOL (The PyMOL Molecular Graphics System, Version 2.0 Schrödinger, LLC). To relax the structure and relieve any clashes arising from the piece-wise modelling and assembly approach taken to generating the *B. burgdorferi* RNA polymerase core model, the assembled complex was subjected to minimization and limited molecular dynamics simulation with the program NAMD^70^ using the CHARMM36m force field^71^. The model was solvated with TIP3P waters and NaCl added to 250 mM using VMD^72^. The system was then minimized using 5,000 steps of conjugate gradient minimization with NAMD ahead of 100 ps of dynamics with a 2 fs integration time step during which the protein was fixed. Langevin dynamics temperature and Nosé-Hoover Langevin piston pressure controls were used to maintain the system at 310 K and 1 atm, respectively, and electrostatic interactions were calculated using the particle-mesh Ewald method. The system was then subjected to a second round of 5,000 steps of conjugate gradient minimization prior to performing 1 ns of unconstrained dynamics in the NPT ensemble. Protein coordinates from frames written every 2 ps over the final 500 ps were positionally-averaged, and bond lengths and angles were subsequently idealized with Rosetta^73^ to generate the final model.

#### Genetic transformation

A C-terminal 10xHis affinity tag was introduced to the chromosomal copy of *rpoC* in the B31–5A4 strain background by homologous recombination. The 3′ end of the *rpoC* gene was amplified using primers encoding a 10xHis affinity tag optimized for *B. burgdorferi* codon usage (rpoC 3285 F: TGCATCTTATGTATTACCAG and rpoC 4131 R + H + AaAg: ACCGGTACTGACGTCTCACTAGTGATGATGATGGTGATGATGGTGATG ATGAACTTCAGAATCGATATTT). The PCR product was TOPO-cloned into the pCR2.1-TOPO vector (Thermo Fisher Scientific, Grand Island, NY, United States). The genomic sequence downstream of *rpoC* was amplified by PCR using the primers rpsL U149F + AatII: GACGTCTGGACATTTAATTCCTACTG and rpsG 385 R + AgeI: ACCGGTATGCATTTAAAAGTTCGTTT. The PCR product encoding the downstream region and the pCR2.1-TOPO-RpoC-10XHis vector were digested by AatII and AgeI restriction enzymes and ligated together with T4 DNA ligase (Invitrogen, Carlsbad, CA, United States). The selectable marker *flgBp*-*aaaC1*-*trpLt*^12^ was inserted on the 3′ end of *rpoC*−10XHis. AatII restriction digest of the pCR2.1-TOPO-RpoC-10xHis vector, and the PCR product encoding the selectable marker was ligated with T4 ligase to generate the plasmid used for homologous recombination. *B. burgdorferi* B31–5A4 strain was transformed with pCR2.1-TOPO-RpoC-10xHis plasmid as described using 30 µg · ml^−1^ gentamicin for selection^74^.

#### RNA polymerase purification

The *B. burgdorferi* B31–5A4-RpoC-10xHis strain was maintained under microaerobic conditions (5% CO_2_, 3% O_2_) in BSK II medium, pH 7.6 at 34 °C. Cultures were passaged in 3 L BSK II media and allowed to reach a cell density of 3–5 × 10^7^ cells · ml^−1^. Cells were collected by centrifugation at 10,000 × *g* for 30 min and then washed once in HN buffer (10 mM HEPES, 10 mM sodium chloride, pH 8.0) to remove residual BSK II. Cell pellets were resuspended in 20 ml of ice-cold lysis buffer containing 50 mM sodium phosphate, 300 mM NaCl, 10 mM imidazole, 2 mM DTT, 5X protease inhibitor cocktail (Invitrogen, Carlsbad, CA, United States), and 200 U · ml^−1^ Turbonuclease (Sigma-Aldrich, St. Louis, MO, United States). Cells were lysed by pushing the cell suspension through a 1-inch diameter French Pressure Cell twice at 3,000 PSI. Cell-free lysates were generated by removing insoluble cell debris from the lysed cell suspension with centrifugation at 4 °C, 20,000 × *g* for 30 min and then filtering the resulting supernatant through a 0.45 µm pore size syringe filter. His-tagged proteins were separated by nickel affinity chromatography. Lysates were loaded into a UPC-900 FPLC (Amersham Biosciences, Little Chalfont, United Kingdom) and pumped through a HisTrapFF 1 mL (GE Healthcare, Chicago, IL, United States) column. The column was washed with 9 mL of 5% elution buffer (50 mM sodium phosphate, 300 mM NaCl, 250 mM imidazole, and 2 mM DTT) and 6 mL 10% elution buffer. The resin-bound proteins were collected into elution fraction by increasing the gradient of elution buffer. The liquid volume of the RNA polymerase containing elution fractions were reduced by filtration in Amicon Ultra – 4 (Millipore, Burlington, MA, United States) 10 kDa pore size centrifugal filter columns. RNA polymerase was subjected to buffer exchange into a storage buffer containing 40 mM HEPES, 200 mM NaCl, and 2 mM DTT with a PD10 Sephadex G-25 column (GE Healthcare, Chicago, IL, United States). The liquid volume was reduced to concentrate RNA polymerase on a centrifugal filter column, and final protein concentration was measured by spectrophotometry to determine A_280nm_ without extinction coefficient adjustment (1 abs at 1 cm = 1 mg · ml^−1^) and BCA assay. The RNA polymerase was stored in 50% glycerol at −80 °C.

#### Sigma factor purification

Oligonucleotides encoding the codon-optimized version of *B. burgdorferi rpoD* were commercially synthesized and cloned into the BamHI/EcoRI site (GenScript, Piscataway, NJ) of the pMAL-C5X plasmid expression vector (New England Biosciences, Ipswich, MA, United States). The expression vector was transformed into Top Shot BL21 (DE3) pLysS Chemically Competent *E. coli* (Invitrogen, Carlsbad, CA, United States) to produce N-terminal Maltose binding protein (MBP)-tagged RpoD. Overnight *E. coli* cultures were passaged 1:200 into LB-Lennox broth containing 2 g · L^−1^ glucose and 100 µg · ml^−1^ ampicillin and then incubated at 32 °C until culture density reached optical density (OD 600 nm) of 0.5. The culture was incubated for an additional 2 hours with 0.3 mM isopropyl β-D-1-thiogalactopyranoside to allow protein expression under the *lac* operator. MBP-tagged proteins were purified from total cell extracts by amylose resin affinity chromatography as described in the pMAL-C5X protein expression system protocols (New England Biosciences, Ipswich, MA, United States). To remove the MBP-tag from the RpoD, 30 mg of purified protein was incubated over night with 200 µg Factor Xa protease in the presence of 2 mM calcium chloride. The mixture containing RpoD was separated by heparin affinity chromatography by flowing the mixture though a HiTrap Heparin HP column. Elution of the column with an increasing gradient of sodium chloride led to the release of recombinant RpoD to apparent homogeneity. RpoD was prepared for storage and use as described under the RNA polymerase purification section.

#### Protein identification

The purified RNA polymerase mixture was separated by SDS-PAGE. Protein bands stained with Imperial Protein Stain (Thermo Fisher Scientific, Grand Island, NY, United States) were excised for analysis by LC/MS/MS at the Research Technology Branch, NIAID, NIH, (Bethesda, MD, United States). Following trypsin gel digest, protein samples were injected onto an Orbitrap Tribrid Mass Spectrometer equipped with Nano-LC Nano-Electrospray source (Thermo Fisher Scientific, Grand Island, NY, United States). Data were analyzed with PEAKS v8.5 (Bioinformatics Solutions Inc. Waterloo, ON, Canada) to discover sequences matching proteins encoded in the *B. burgdorferi* B31 genome. Sequence matches were collated, and protein identities were ranked based on highest number of sequence matches normalized to predicted protein size.

#### Quantitative western blots

Custom polyclonal antibodies were generated to full-length recombinant *Borrelia* RNA polymerase subunits β, α and σ^70^ (RpoD) (GenScript, Piscataway, NJ, United States). Linear detectable ranges of the polyclonal antibodies anti-RpoB, anti-RpoA, and anti-RpoD were determined by linear regression analysis. Analysis was performed on densitometry signals resulting from western blots loaded with twofold dilutions of recombinant target proteins and incubated with 1:2000 dilution of primary antibodies for 16 hours. Purified RNA polymerase was loaded in amounts to produce densitometry signals within the linear range (100–150 ng of purified protein) to quantify the RNA polymerase subunits purified by affinity chromatography. For western blotting, proteins were separated by SDS-PAGE in the Mini-Tetra Gel System (Bio-Rad, Hercules, CA, United States) and transferred to PVDF membrane using the Transblot Turbo System (Bio-Rad, Hercules, CA, United States). Primary antibodies were incubated with the membrane at a 1:2000 dilution for 17 hours. The antibodies bound to antigen were labeled by incubation of the membrane with HRP-conjugated protein-A (Invitrogen, Carlsbad, CA, United States) at a 1:4000 dilution for one hour and then the membrane was subjected to five 15-min washing steps in TBST. Labeled antibodies were detected by soaking the membrane with Super Signal West Pico chemiluminescent substrate kit (Thermo Fisher Scientific, Grand Island, NY, United States) on the ChemiDoc MP imaging system (Bio-Rad, Hercules, CA, United States). Chemiluminescent signals were quantified from ChemiDoc images by densitometry in Image Lab Software (Bio-Rad, Hercules, CA, United States).

#### Generation of templates for in vitro transcription

A single-stranded circular DNA template was generated for RNA polymerase activity (without sigma factor). A 45 bp oligonucleotide NC-45 (CTGGAGGAGATTTTGTGGTATCGATTCGTCTCTTAGAGGAAGCTA) was combined with splint oligonucleotide (CTCCAGTAGCTT) to a promote double-stranded complex and phosphodiester bond formation by T4 DNA ligase, resulting in single-stranded NC-45 oligonucleotide circularization^75^. Linear dsDNA templates were generated to detect sigma factor-dependent transcription from *B. burgdorferi* promoters. Transcriptional start sites upstream of genes of interest that were previously identified by either primer extension or RNAseq were targeted for template generation^32,41,43,76^. Primers were generated to amplify a 500-bp region that contains the previously annotated transcriptional start site in the middle of the amplicon (Table S2). DNA was amplified by PCR with Q5 High Fidelity Polymerase (New England Biosciences, Ipswich, MA, United States) from *B. burgdorferi* strain B31-A3 genomic DNA. PCR products were purified using the QIAquick PCR purification kit (QIAGEN, Germantown, MD, United States).

### *In vitro* transcription

The *in vitro* transcription reactions were carried out under the following conditions unless otherwise stated. A 5X reaction buffer containing 300 mM potassium glutamate, 200 mM HEPES pH 7.5, 5 mM DTT, 0.25% NP40 detergent, 10 mM MgSO_4_, and 25 mM MnSO_4_ in HPLC grade water were stored for up to two weeks at −80 °C and mixed to a 1X concentration in the reaction mixture. The final reaction mixture contained 1X reaction buffer, 0.8 U RiboLock RNase inhibitor (Invitrogen, Carlsbad, CA, United States), 21 nM RNA polymerase, 500 nM RpoD, 2 µCi ATP [α-^32^P] (PerkinElmer, Waltham, MA, United States), 20 µM ATP, 200 µM GTP, 200 µM CTP, and 200 µM UTP. A preliminary mixture containing reaction buffer, RiboLock RNase inhibitor (Invitrogen, Carlsbad, CA, United States), RNA polymerase, and RpoD was incubated for 10 min on ice prior to the addition of other components. Transcription was initiated with the addition of linear dsDNA template to a concentration of 10 nM, and reactions were allowed to proceed for 5 min at 37 °C. RNA products were separated by gel electrophoresis in 10% TBE-urea gels (Invitrogen, Carlsbad, CA, United States) at 180 V for 45 min. To detect accumulation of RNA that incorporated α-^32^P-ATP, gels were placed on a Phosphor Screen (GE Healthcare, Chicago, IL, United States) overnight (16 hours), and the resulting signal was detected using the Typhoon FLA 9500 (GE Healthcare, Chicago, IL, United States). Densitometry measurements were determined with Image Lab 6.0.1. Software (Bio-Rad, Hercules, CA, United States).

## Supplementary information

Supplementary information.
