# Supplementary material for: Establishment of an in vitro RNA polymerase transcription system: a new tool to study transcriptional activation in Borrelia burgdorferi
Source: Sci Rep. 2020 May 19;10:8246. doi: 10.1038/s41598-020-65104-y (PMC7237435; doi:10.1038/s41598-020-65104-y)

**Supplementary Figures and Tables**

Establishment of an *in vitro* RNA polymerase transcription system: a new tool to study transcriptional activation in *Borrelia burgdorferi*

**William K. Boyle^1,2^, Laura S. Hall^3^, Anthony A. Armstrong^4^, Daniel P. Dulebohn^2^, D. Scott Samuels^3^, Frank C. Gherardini^2^, Travis J. Bourret^1^**

*To whom correspondence should be addressed: Travis J. Bourret: Department of Medical Microbiology and Immunology, Creighton University, 2500 California Plaza, Omaha, NE 68178; E-mail: [TravisBourret@creighton.edu](mailto:TravisBourret@creighton.edu); Tel.(402) 280-3750; Fax.(402) 280-1875.


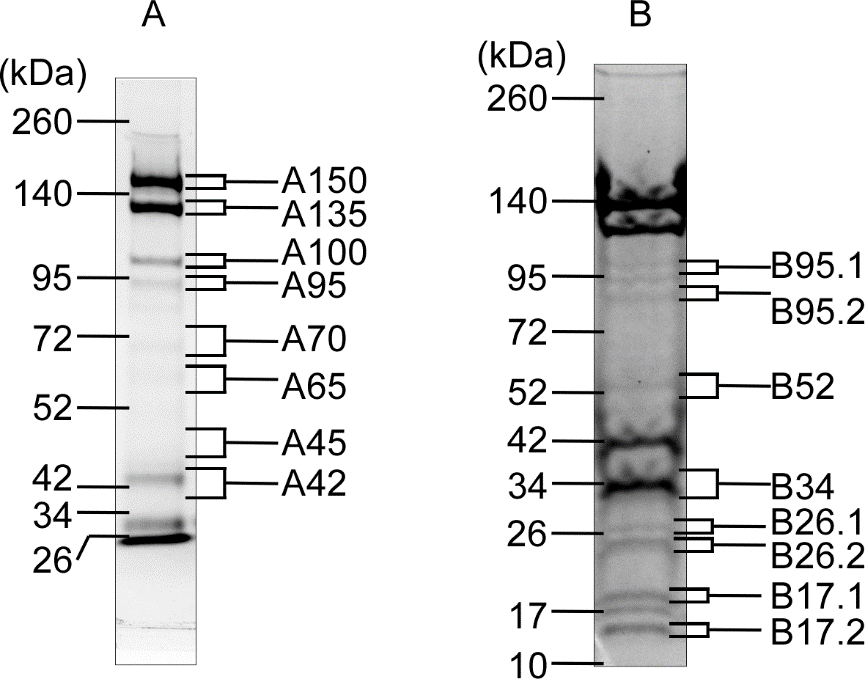


**Figure S1**. Sample labeling for mass spectrometry. See Supplemental Table S1 for peptides identified by mass spectrometry.


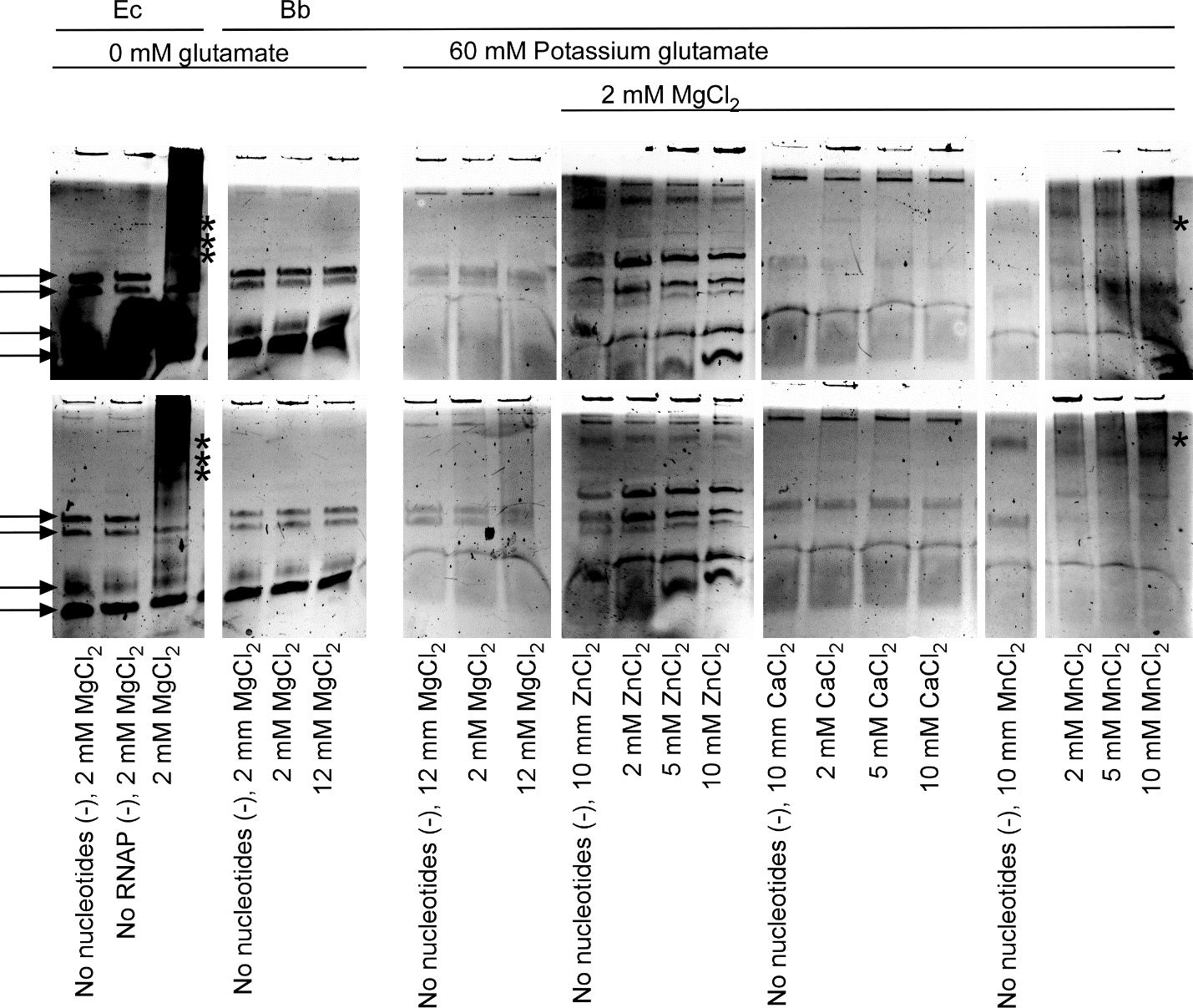


**Figure S2**. Screening for cofactors required for *B. burgdorferi* RNAP activity reveals a role for Mn^2+^. RNA and DNA were separated by gel electrophoresis and detected by incorporation of SYBR Safe dye. Two representative gels are shown for each reaction condition. Top label indicates RNA polymerase from *E. coli* (Ec) or *B. burgdorferi* (Bb) species added to the reaction. Reaction conditions utilized for multiple reactions are indicated above the gels. Concentrations of divalent metal cations for each reaction are indicated below the gels. Arrows indicate migration of circular single stranded DNA templates and asterisks (*) indicate dye incorporation into RNA products.

**Table S1**. LC-MS identified peptides represented in each sample excised from RNA polymerase mixture separated by SDS-PAGE.

| **Sample Location** | **Protein ID** | **Description** | **Protein Accessions** | **Number of matches** | **Coverage (%)** | **Number of unique matches** |
| --- | --- | --- | --- | --- | --- | --- |
| A150 | RpoC | DNA-directed RNA polymerase subunit beta' | recombRPOC | 599 | 70 | 123 |
| A135 | RpoB | DNA-directed RNA polymerase subunit beta | Q59191 | 516 | 74 | 110 |
| A135 | RpoC | DNA-directed RNA polymerase subunit beta' | recombRPOC | 108 | 50 | 69 |
| A135 | Tuf | Elongation factor Tu | P50062 | 2 | 5 | 2 |
| A100 | RpoB | DNA-directed RNA polymerase subunit beta | Q59191 | 76 | 43 | 45 |
| A100 | RpoC | DNA-directed RNA polymerase subunit beta' | recombRPOC | 43 | 32 | 41 |
| A100 | BB0536 | Zinc protease putative | O51486 | 29 | 26 | 24 |
| A100 | SecA | Protein translocase subunit SecA | O07497 | 19 | 22 | 19 |
| A100 | HrpA | ATP-dependent helicase HrpA | O51767 | 9 | 11 | 9 |
| A95 | RpoC | DNA-directed RNA polymerase subunit beta' | recombRPOC | 52 | 29 | 43 |
| A95 | RpoD | RNA polymerase sigma factor RpoD | P52323 | 49 | 40 | 25 |
| A95 | P83/P100 | Borrelia P83/P100 antigen | Q45013 | 22 | 26 | 21 |
| A95 | RpoB | DNA-directed RNA polymerase subunit beta | Q59191 | 15 | 11 | 13 |
| A95 | GreA | Transcription elongation factor GreA | O51157 | 8 | 9 | 8 |
| A70 | GroL | 60 kDa chaperonin | P0C923 | 203 | 66 | 36 |
| A70 | RpoC | DNA-directed RNA polymerase subunit beta' | recombRPOC | 52 | 21 | 23 |
| A70 | NusA | Transcription termination/antitermination protein | O51740 | 50 | 39 | 19 |
| A70 | P66 | Integral outer membrane protein P66 | H7C7N8 | 37 | 43 | 19 |
| A70 | RpoB | DNA-directed RNA polymerase subunit beta | Q59191 | 12 | 10 | 11 |
| A65 | RpoC | DNA-directed RNA polymerase subunit beta' | recombRPOC | 64 | 16 | 24 |
| A65 | BB0330 | Bacterial extracellular solute-binding protein family 5 | O51308 | 49 | 30 | 19 |
| A65 | OppA4 | Oligopeptide ABC transporter | H7C7K8 | 36 | 29 | 18 |
| A65 | BB0328 | Bacterial extracellular solute-binding protein family 5 | O51307 | 38 | 19 | 10 |
| A65 | BB0243 | Glycerol-3-phosphate dehydrogenase | O51259 | 27 | 28 | 15 |
| A45 | Tuf | Elongation factor Tu | P50062 | 110 | 57 | 23 |
| A45 | RpoC | DNA-directed RNA polymerase subunit beta' | recombRPOC | 31 | 18 | 26 |
| A45 | PfkA | ATP-dependent 6-phosphofructokinase | O51669 | 32 | 47 | 22 |
| A45 | BBK32 | Fibronectin-binding protein | O50835 | 20 | 36 | 13 |
| A45 | Csd | Probable cysteine desulfurase | O51111 | 22 | 44 | 14 |
| A42 | RpoA | DNA-directed RNA polymerase subunit alpha | O51455 | 134 | 57 | 30 |
| A42 | FlaB | Flagellar filament 41 kDa core protein | P11089 | 30 | 47 | 14 |
| A42 | Tuf | Elongation factor Tu | P50062 | 36 | 46 | 18 |
| A42 | BmpA | Basic membrane protein A | Q45010 | 17 | 36 | 10 |
| A42 | FlaA | Flagellar filament outer layer protein | P70856 | 7 | 21 | 7 |
| B95.1 | P83/P100 | Borrelia P83/P100 antigen | Q45013 | 83 | 64 | 54 |
| B95.1 | RpoD | RNA polymerase sigma factor RpoD | P52323 | 60 | 51 | 34 |
| B95.1 | BB0536 | Zinc protease putative | O51486 | 87 | 53 | 57 |
| B95.1 | GreA | Transcription elongation factor GreA | O51157 | 79 | 60 | 56 |
| B95.1 | DnaK | Chaperone protein DnaK | P0C922 | 58 | 58 | 34 |
| B95.2 | GuaA | GMP synthase [glutamine-hydrolyzing] | P0CL64 | 75 | 57 | 31 |
| B95.2 | GroL | 60 kDa chaperonin | P0C923 | 108 | 67 | 42 |
| B95.2 | BB0330 | Bacterial extracellular solute-binding protein family 5 | O51308 | 81 | 64 | 42 |
| B95.2 | DnaK | Chaperone protein DnaK | P0C922 | 88 | 63 | 42 |
| B95.2 | P66 | Integral outer membrane protein P66 | H7C7N8 | 77 | 62 | 38 |
| B52 | RpoA | DNA-directed RNA polymerase subunit alpha | O51455 | 688 | 87 | 61 |
| B52 | GuaA | GMP synthase [glutamine-hydrolyzing] | P0CL64 | 234 | 72 | 58 |
| B52 | RpoC | DNA-directed RNA polymerase subunit beta' | recombRPOC | 185 | 59 | 96 |
| B52 | BB0713 | Uncharacterized protein | O51655 | 351 | 65 | 22 |
| B52 | GroL | 60 kDa chaperonin | P0C923 | 138 | 74 | 50 |
| B34 | Ldh | L-lactate dehydrogenase | O51114 | 621 | 97 | 113 |
| B34 | BB0713 | Uncharacterized protein | O51655 | 1061 | 85 | 51 |
| B34 | OspA | Outer surface protein A | P0CL66 | 519 | 79 | 61 |
| B34 | ThyX | Flavin-dependent thymidylate synthase | O50965 | 552 | 60 | 35 |
| B34 | RpsD | 30S ribosomal protein S4 | O51560 | 70 | 63 | 23 |
| B26.1 | RpsD | 30S ribosomal protein S4 | O51560 | 149 | 73 | 38 |
| B26.1 | RplC | 50S ribosomal protein L3 | P94267 | 322 | 81 | 33 |
| B26.1 | OspA | Outer surface protein A | P0CL66 | 155 | 75 | 45 |
| B26.1 | Ndk | Nucleoside diphosphate kinase | O51419 | 70 | 69 | 25 |
| B26.1 | Ldh | L-lactate dehydrogenase | O51114 | 58 | 72 | 31 |
| B26.2 | RpsD | 30S ribosomal protein S4 | O51560 | 144 | 79 | 49 |
| B26.2 | Ndk | Nucleoside diphosphate kinase | O51419 | 108 | 87 | 38 |
| B26.2 | Crr | PTS system glucose-specific EIIA component | Q44840 | 63 | 91 | 30 |
| B26.2 | P22 | Outer surface 22 kDa lipoprotein | P0CL67 | 61 | 79 | 20 |
| B26.2 | BB0449 | Uncharacterized protein | O51405 | 41 | 42 | 9 |
| B17.1 | Ndk | Nucleoside diphosphate kinase | O51419 | 379 | 92 | 61 |
| B17.1 | BB0449 | Uncharacterized protein | O51405 | 58 | 42 | 13 |
| B17.1 | P22 | Outer surface 22 kDa lipoprotein | P0CL67 | 54 | 74 | 18 |
| B17.1 | RplM | 50S ribosomal protein L13 | O51314 | 28 | 45 | 13 |
| B17.1 | BBA03 | Putative outer membrane protein | Q44849 | 46 | 70 | 19 |
| B17.2 | BB0449 | Uncharacterized protein | O51405 | 149 | 61 | 22 |
| B17.2 | Ndk | Nucleoside diphosphate kinase | O51419 | 98 | 75 | 30 |
| B17.2 | RplQ | 50S ribosomal protein L17 | O51456 | 55 | 60 | 18 |
| B17.2 | CspA | Complement regulator-acquiring surface protein 1 | O50957 | 3 | 10 | 3 |
| B17.2 | BB0047 | Uncharacterized protein | O51076 | 28 | 60 | 10 |
| B17.3 | BB0449 | Uncharacterized protein | O51405 | 202 | 79 | 28 |
| B17.3 | RpoB | DNA-directed RNA polymerase subunit beta | Q59191 | 186 | 64 | 94 |
| B17.3 | RpoC | DNA-directed RNA polymerase subunit beta' | recombRPOC | 148 | 53 | 87 |
| B17.3 | BB0034 | Outer membrane protein P13 | H7C7R6 | 28 | 46 | 13 |
| B17.3 | SpoVG | Putative septation protein SpoVG | O51726 | 18 | 56 | 12 |

**Table S2.** Oligonucleotides used for *in vitro* transcription template generation.

| **Primer** | **Sequence (5′ to 3′)** |
| --- | --- |
| *rrlBp* F | GGACGAACCTCTAGTGTACCAG |
| *rrlBp* R | TTTATCTTCCATCTCTATTTTGCC |
| *napAp* F | TGCATTGGGGTTGTGCC |
| *napAp* R | TTTTGTGAATAACAAAGAAATTGGTATC |
| *ospAp* F | AATCAAGACAAACATTGCTGC |
| *ospAp* R | GCAATTAGATCGTACTTGCCG |
| *dbpBp* F | GCAAAATAACCAATTTGAAATATTTTGGC |
| *dbpBp* R | TTCAAAAAGTACACCTTTTCCCG |
| *gapdhp* F | GGATTATGTTCAAGAAGGAGCC |
| *gapdhp* R | CACAACAATGGCACCATCT |
| *enop* F | TGATCAAAGTCAAAGGCAGC |
| *enop* R | CTAAGCTCAACAGCCTCG |
| *groLp* F | GGAAATGTCCAATGTATTTTGTAAGC |
| *groLp* R | CCATCCTTTGTAACCGTTGG |
| *bbd18p* F | CTTATCTATATTAGAAGCCTCTACCG |
| *bbd18p* R | TTCTTTAAACTTATGGGAATTTTAAAAAAGTC |
| *flgBp* F | TGATTTAGATTTAAAGTTTAGTGAGG |
| *flgBp* R | TTTAAACCCAGACAGGTGC |
| *clpCp* F | AAAACTTTGAGGGGGCC |
| *clpCp* R | CTCTAATTTATCTATTTCAGATATAACTTCTTG |
| *rplUp* F | TGAGCTTGAATATGGTTTTGGG |
| *rplUp* R | AGGTACATCTAATAAGAGAATTTACGAC |
| *uvrBp* F | GTATGTAATTGCATATTCCTTGGC |
| *uvrBp* R | TTTCCACTGCCTGTAACACC |
| *rpoSp* F | CTTGGAGGAAATTGATGGAAACC |
| *rpoSp* R | TCTCTTACTGATTTTAAATATATGTTTAAATCCTC |
| *nagAp* F | TTCTATTAATTAACTGCCAGGCAC |
| *nagAp* R | AAGTCTGTCAGACGTTACAATATC |
| *glpFp* F | AAGGAACTTCCAACTCCTTTG |
| *glpFp* R | CTTGGAATTTTGTATAATTCATAATTATATCTCC |
| *rpoDp* F | TTTATTTGAAAATAATAAAGATTTTTCATTAATGG |
| *rpoDp* R | CCAACCTTATTCCCCTATCCTC |
| *rpoNp* F | AATTAAACTTTCCTTCCGGGC |
| *rpoNp* R | TGGAAGGCAAAATTCCTGC |
| *ospCp* F | CGCCAATTTCTCTAATTCTTCTTGC |
| *ospCp* R | GCTTCAACCTCTTTCACAGC |

**Table S3. Alignment of RpoC amino acid sequences.**

*Borrelia* MK-------------EIKDFERIKIKIASPDQIRNWSYGEVKKSETINYRTLRPEKDGLFCERIFGTTKEWECYCGKFKSVRYKGIICDRCNVEVTHFKV

*Staphylococcus* ----------------------MKIGLASPEKIRSWSFGEVKKPETINYRTLKPEKDGLFCERIFGPTKDWECSCGKYKRVRYKGMVCDRCGVEVTKSKV

*Bacillus* ML-------------DVNNFEYMNIGLASPDKIRSWSFGEVKKPETINYRTLKPEKDGLFCERIFGPTKDWECHCGKYKRVRYKGVVCDRCGVEVTRAKV

*Clostridioides* MF-------------ELNNFESIKIALASPEKIRQWSRGEVKKPETINYRTLKPEKDGLFCERIFGPQKDWECHCGKYRRVRYKGVVCDRCGVEVTKSKV

*Rhodospirillum* MNE-LM--KIFGQVSGTQAFDQIKISIASPEKIRSWSFGEIKKPETINYRTFKPERDGLFCARIFGPIKDYECLCGKYKRMKYRGIICEKCGVEVTLSKV

*Rhodobacter* MNQEITNNNPFNPLAQPKAFDEIKISLASPERILSWSYGEIKKPETINYRTFKPERDGLFCARIFGPIKDYECLCGKYKRMKYRGVVCEKCGVEVTLQKV

*Caulobacter* MNQEVL--NIFNPVQAAPTFDQIRISLASPEKIRSWSFGEIKKPETINYRTFKPERDGLFCARIFGPTKDYECLCGKYKRMKYKGIICEKCGVEVTLARV

*Pseudomonas* MKD-LL--NLLKNQGQIEEFDAIRIGLASPEMIRSWSFGEVKKPETINYRTFKPERDGLFCAKIFGPVKDYECLCGKYKRLKHRGVICEKCGVEVALAKV

*Azotobacter* MKD-LL--NLLKNQGQLEEFDAIRIGLASPEMIRSWSFGEVKKPETINYRTFKPERDGLFCAKIFGPVKDYECLCGKYKRLKHRGVICEKCGVEVALAKV

*Escherichia* MKD-LL--KFLKAQTKTEEFDAIKIALASPDMIRSWSFGEVKKPETINYRTFKPERDGLFCARIFGPVKDYECLCGKYKRLKHRGVICEKCGVEVTQTKV

*Mycolicibacterium* ML-------------DVNFFDELRIGLATADDIRNWSYGEVKKPETINYRTLKPEKDGLFCEKIFGPTRDWECYCGKYKRVRFKGIICERCGVEVTRAKV

*Borrelia* RRERMGHIELAAPVAHIWYYKYIPSRIGLLLDITASSLNSILYYEKYVVIEPG-----------------------------------------------

*Staphylococcus* RRERMGHIELAAPVSHIWYFKGIPSRMGLLLDMSPRALEEVIYFASYVVVDPG-----------------------------------------------

*Bacillus* RRERMGHIELAAPVSHIWYFKGIPSRMGLVLDMSPRALEEVIYFASYVVTDPA-----------------------------------------------

*Clostridioides* RRERMGHIELAAPMSHIWYFKGIPSRMGLLLDMSPRSLEKILYFASYVVVDPG-----------------------------------------------

*Rhodospirillum* RRERMGHIELAAPVAHIWFMKSLPSRVGLLIDMTLKDLERVLYFENYVVVEPG-----------------------------------------------

*Rhodobacter* RRERMGHIELASPVAHIWFLKSLPSRIGLMLDMTLRDLERILYFENYVVIEPG-----------------------------------------------

*Caulobacter* RRERMGHIELASPVAHIWFLKSLPSRIAMMLDMPLKDIERVLYFEYYIVTEPG-----------------------------------------------

*Pseudomonas* RRERMGHIELASPVAHIWFLKSLPSRIGLLLDMTLRDIERVLYFESYVVIDPG-----------------------------------------------

*Azotobacter* RRERMAHIELASPVAHIWFLKSLPSRIGLLLDMTLRDIERVLYFESYVVIDPG-----------------------------------------------

*Escherichia* RRERMGHIELASPTAHIWFLKSLPSRIGLLLDMPLRDIERVLYFESYVVIEGG-----------------------------------------------

*Mycolicibacterium* RRERMGHIELAAPVTHIWYFKGVPSRLGYLLDLAPKDLEKIIYFAAYVITSVDDEMRHNELSTLEAEMAVEKKAVEDQRDADLEARAQKLEADLAELEAE

*Borrelia* --------------------------------------DTDLKKMQLLN-EDEYIEARERYGM-SFNASMGAEAIKTLLENLDLDELSSKLRIQMIDKDD

*Staphylococcus* --------------------------------------PTGLEKKTLLS-EAEFRDYYDKYPG-QFVAKMGAEGIKDLLEEIDLDEELKLLRDELESATG

*Bacillus* --------------------------------------NTPLEKKQLLS-EKEYRAYLDKYGN-KFQASMGAEAIHKLLQDIDLVKEVDMLKEELKTSQG

*Clostridioides* --------------------------------------ETGLNEKQLLT-EKEYRTALEKYGY-TFTVGMGAEAVKTLLQNIDLEQQSKDLRAELKDSTG

*Rhodospirillum* --------------------------------------LTPLKLHEMLS-EEQYQRAVEEYGEDSFTAGIGAEAIRDMLMSIDLETLKTDMKVELRDTTS

*Rhodobacter* --------------------------------------LTELSYGQLLT-EDEYLDAQDQFGADAFTANIGAEAIREMLSAIDLEATAEQLREELKEATG

*Caulobacter* --------------------------------------LTPLKQHQLLS-EDDYMRAQEEYGDDSFTAEIGAEAIQNLLKAIDLEKEAERLREELSGTVS

*Pseudomonas* --------------------------------------MTTLEKGQLLN-DEQYFEALEEFGD-DFDARMGAEAVHELLNAIDLEHEIGRLREEIPQTNS

*Azotobacter* --------------------------------------MTTLEKGQLLN-DEQYFEALEEFGD-DFDARMGAEAVRELLNQIDLEHEIGRLREEIPQTNS

*Escherichia* --------------------------------------MTNLERQQILT-EEQYLDALEEFGD-EFDAKMGAEAIQALLKSMDLEQECEQLREELNETNS

*Mycolicibacterium* GAKSDVRRKVRDSGEREMRQLRDRAQRELDRLDEIWNTFTKLAPKQLIVDEVLYRELQDRYGE-YFTGAMGAESIKKLIENFDIDAEAESLREVIRSGKG

*Borrelia* KT-DKKLLRRLEIIENFKISGNKPEWMIMEVLPVIPPEIRPMVQLDGGRFATSDLNDLYRRVINRNNRLRKLLLLNAPEIIVRNEKRMLQESVDSLFDNS

*Staphylococcus* QR-LTRAIKRLEVVESFRNSGNKPSWMILDVLPIIPPEIRPMVQLDGGRFATSDLNDLYRRVINRNNRLKRLLDLGAPGIIVQNEKRMLQEAVDALIDNG

*Bacillus* QR-RTRAIKRLEVLEAFRNSGNKPSWMILDVLPVIPPELRPMVQLDGGRFATSDLNDLYRRVINRNNRLKRLLDLGAPSIIVQNEKRMLQEAVDALIDNG

*Clostridioides* QK-KVRTIRRLEVVEAFKKSGNKPEWMILDAIPVIPPDLRPMVQLDGGRFATSDLNDLYRRVINRNNRLKRLLELGAPDIIVRNEKRMLQEAVDALIDNG

*Rhodospirillum* EAKRKKLVKRLKIVDAFIESGCRPEWMILEVIPVIPPELRPLVPLDGGRFATSDLNDLYRRVINRNNRLKRLIELRAPDIIIRNEKRMLQESVDALFDNG

*Rhodobacter* ELKPKKIIKRLKIVESFLESKNRPEWMILTVLPVIPPELRPLVPLDGGRFATSDLNDLYRRVINRNNRLKRLIELRAPDIIVRNEKRMLQEAVDALFDNG

*Caulobacter* DMKQKKFSKRLKILEAFQESGNRPEWMVLTVVPVIPPELRPLVPLDGGRFATSDLNDLYRRVINRNNRLKRLIELRAPDIIIRNEKRMLQESVDALFDNG

*Pseudomonas* ETKIKKLSKRLKLMEAFQGSGNKPEWMVLTVLPVLPPDLRPLVPLDGGRFATSDLNDLYRRVINRNNRLKRLLDLAAPDIIVRNEKRMLQEAVDALLDNG

*Azotobacter* ETKIKKLSKRLKLMEAFQGSGNLPEWMVLTVLPVLPPDLRPLVPLDGGRFATSDLNDLYRRVINRNNRLKRLLDLAAPDIIVRNEKRMLQEAVDALLDNG

*Escherichia* ETKRKKLTKRIKLLEAFVQSGNKPEWMILTVLPVLPPDLRPLVPLDGGRFATSDLNDLYRRVINRNNRLKRLLDLAAPDIIVRNEKRMLQEAVDALLDNG

*Mycolicibacterium* QK-KLRALKRLKVVAAFQQSGNSPMGMVLDAVPVIPPELRPMVQLDGGRFATSDLNDLYRRVINRNNRLKRLIDLGAPEIIVNNEKRMLQESVDALFDNG

*Borrelia* HKRKVVKGSSSRPLKSLSDALKGKQGRFRQNLLGKRVDYSGRSVIVVGPELKLHQCGLPAKMALELFKPFVIRRLIESEAVFNIKRAKNLIEQEVDEVWQ

*Staphylococcus* RRGRPVTGPGNRPLKSLSHMLKGKQGRFRQNLLGKRVDYSGRSVIAVGPSLKMYQCGLPKEMALELFKPFVMKELVQREIATNIKNAKSKIERMDDEVWD

*Bacillus* RRGRPVTGPGNRPLKSLSHMLKGKQGRFRQNLLGKRVDYSGRSVIVVGPHLKMYQCGLPKEMALELFKPFVMKELVEKGLAHNIKSAKRKIERVQPEVWD

*Clostridioides* RRGRPVTGPGNRPLKSLSDMLKGKQGRFRQNLLGKRVDYSGRSVIVVGPELKFYQCGLPKKMALELFKPFVMDKLVKEGYAHNIKSAKSIVEKVKPEVWD

*Rhodospirillum* RRGRAITGANKRPLKSLSDMLKGKQGRFRQNLLGKRVDYSGRSVIVVGPELKLHQCGLPKKMALELFKPFVYSKLEQYHYATTIKAAKRMVEKERPEVWD

*Rhodobacter* RRGRVITGTNKRPLKSLSDMLKGKQGRFRQNLLGKRVDFSGRSVIVTGPELKLHQCGLPKKMALELFKPFIYSRLEAKGLSSTVKQAKKLVEKERPEVWD

*Caulobacter* RRGRVITGANKRPLKSLADMLKGKQGRFRQNLLGKRVDYSGRSVIVVGPELKLHECGLPKKMALELFKPFIYARLDAKGLSGTVKQSKRMVEREQPQVWD

*Pseudomonas* RRGRAITGSNKRPLKSLADMIKGKQGRFRQNLLGKRVDYSGRSVITVGPTLRLHQCGLPKKMALELFKPFIFGKLEGRGMATTIKAAKKMVERELPEVWD

*Azotobacter* RRGRAITGSNKRPLKSLADMIKGKQGRFRQNLLGKRVDYSGRSVITVGPTLRLHQCGLPKKMALELFKPFIFGKLETRGMATTIKAAKKMVERELPEVWD

*Escherichia* RRGRAITGSNKRPLKSLADMIKGKQGRFRQNLLGKRVDYSGRSVITVGPYLRLHQCGLPKKMALELFKPFIYGKLELRGLATTIKAAKKMVEREEAVVWD

*Mycolicibacterium* RRGRPVTGPGNRPLKSLSDLLKGKQGRFRQNLLGKRVDYSGRSVIVVGPQLKLHQCGLPKLMALELFKPFVMKRLVDLNHAQNIKSAKRMVERQRPQVWD

*Borrelia* ILDLVIKEHPILLNRAPTLHRLGIQAFEPVLVEGKAIKLHPLVCHAYNADFDGDQMAVHVPLTPAAQAESWALMLSTNNLLNPANGHPIVFPSQDIVLGL

*Staphylococcus* VLEEVIREHPVLLNRAPTLHRLGIQAFEPTLVEGRAIRLHPLVTTAYNADFDGDQMAVHVPLSKEAQAEARMLMLAAQNILNPKDGKPVVTPSQDMVLGN

*Bacillus* VLESVIKEHPVLLNRAPTLHRLGIQAFEPTLVEGRAIRLHPLVCTAYNADFDGDQMAVHVPLSAEAQAEARILMLAAQNILNPKDGKPVVTPSQDMVLGN

*Clostridioides* VLEDVIKSHPVLLNRAPTLHRLGIQAFEPILVEGKAIKLHPLVCTAYNADFDGDQMAVHVPLSVEAQAEARFLMLSVNNILAPKDGSPITTPSQDMVLGC

*Rhodospirillum* ILEEVIREHPVMLNRAPTLHRLGIQAFEPVLIEGKAIQLHPLVCTAFNADFDGDQMAVHVPLSLEAQLEARVLMMSTNNILSPANGKPIIVPTQDIVLGL

*Rhodobacter* ILDEVIREHPVLLNRAPTLHRLGIQAFEPTLIEGKAIQLHPLVCSAFNADFDGDQMAVHVPLSLEAQLEARVLMMSTNNVLSPANGSPIIVPSQDMILGL

*Caulobacter* ILEEVIREHPVLLNRAPTLHRLGIQAFEPKLIEGKAIQLHPLVCAAFNADFDGDQMAVHVPLSLEAQLEARVLMMSTNNILSPANGRPIIVPSQDIVLGL

*Pseudomonas* VLAEVIREHPVLLNRAPTLHRLGIQAFEPVLIEGKAIQLHPLVCAAYNADFDGDQMAVHVPLTLEAQLEARALMMSTNNILSPANGEPIIVPSQDVVMGL

*Azotobacter* VLAEVIREHPVLLNRAPTLHRLGIQAFEPVLIEGKAIQLHPLVCAAYNADFDGDQMAVHVPLTLEAQLEARALMMSTNNILSPANGEPIIVPSQDVVLGL

*Escherichia* ILDEVIREHPVLLNRAPTLHRLGIQAFEPVLIEGKAIQLHPLVCAAYNADFDGDQMAVHVPLTLEAQLEARALMMSTNNILSPANGEPIIVPSQDVVLGL

*Mycolicibacterium* VLEEVIAEHPVLLNRAPTLHRLGIQAFEPQLVEGKAIQLHPLVCEAFNADFDGDQMAVHLPLSAEAQAEARILMLSSNNILSPASGKPLAMPRLDMVTGL

*Borrelia* YYLTMEKK-NVVGEGK----------KFLNFNNVILAINNRSLDYNASIYVKIHG---------------------EYKKTTAGRVIFNEALPKG--IEF

*Staphylococcus* YYLTLERK-DAVNTGA----------IFNNTNEVLKAYANGFVHLHTRIGVHASSFNN---------PTFTEEQNKKILATSVGKIIFNEIIPDS--FAY

*Bacillus* YYLTLERA-GAVGEGM----------VFKNTDEALLAYQNGYVHLHTRVAVAANSLKN---------VTFTEEQRSKLLITTVGKLVFNEILPES--FPY

*Clostridioides* YYLTIEAQDGAKGTGM----------VFKDFNELLLAYYNKSVHLHALVKLKVTLE----------------DGRSSLVESTVGRFIFNENIPQD--LGF

*Rhodospirillum* YYLTLDRE-GEKGEGM----------AFASLNEIEHALQARVVSLQARVKARLHTID----------ENGAPV--IRTVETTPGRMLLSRLLPRHTALPF

*Rhodobacter* YYTTMERK-GMVGEGM----------VFASVEEVEHALTSGAVHLHAKVKARVKQID----------EEGNEV--WKRYDTTPGRIRLGALLPLNAKAPF

*Caulobacter* YYLSVARD-GEPGEGK----------IFADLGEIEAAMDAGVVSLHAKIKARHTEMT----------PEGVLL--RKVIDTTPGRMKIAALLPHHPQIGH

*Pseudomonas* YYMTREAI-NAKGEGM----------AFADLQEVDRAYRSGQASLHARVKVRINEKIKG--------EDGQLTANTRIVDTTVGRALLFQVVPAG--LPF

*Azotobacter* YYMTREAV-NARGEGR----------VFADLQEVDRVFRGGEASLHARVKVRINETVRQ--------KDGSLVHNTRIVDTTVGRALLFQVVPAG--LSF

*Escherichia* YYMTRDCV-NAKGEGM----------VLTGPKEAERLYRSGLASLHARVKVRITEYEKD--------ANGELVAKTSLKDTTVGRAILWMIVPKG--LPY

*Mycolicibacterium* YYLTTLVE-GATGEYQAATKDAPEQGVYSSPAEAIMAMDRGALSVRAKIKVRLTELRPPTDLEAQLFENGWKPGDAWTAETTLGRVMFNELLPKS--YPF

*Borrelia* -------------------------------------VNKTLSDLELQILISKVYVVHGSSIVIEMLDIIKELGFRYATKFGCTISMSDIIVPDEKRTYV

*Staphylococcus* INEPTQENLERKTPNRYFIDPTTLGEGGLKEYFENEELIEPFNKKFLGNIIAEVFNRFSITDTSMMLDRMKDLGFKFSSKAGITVGVADIVVLPDKQQIL

*Bacillus* MNEPTKSNIEEKTPDRFFLEKG----ADVKAVIAQQPINAPFKKGILGKIIAEIFKRFHITETSKMLDRMKNLGFKYSTKAGITVGVSDIVVLDDKQEIL

*Clostridioides* VDRKEN---------PFAL-----------------EVDFLADKKSLGKIIDKCFRKHGNTETAELLDYIKALGFKYSTLGGITVAVDDMSVPEEKKVFI

*Rhodospirillum* -----------------------------------SVINRLLRKKDITDVIDTVYRHCGQKETVIFCDRVMQLGYAHAARAGISFGKDDLVIPPTKAQLV

*Rhodobacter* -----------------------------------DLVNRPLRKKDVQNVIDTVYRYCGQKESVIFCDQIMGLGFREAFKAGISFGKDDMLIPERKWEIV

*Caulobacter* -----------------------------------RLIEKALTKKEIGNLIDIVYRHCGQKATVIFADKVMGLGFKEAAKAGISFGKDDIIIPVRKTAIV

*Pseudomonas* -----------------------------------DVVNQSMKKKAISKLINHCYRVVGLKDTVIFADQLMYTGFAYSTISGVSIGVNDFVIPDEKARII

*Azotobacter* -----------------------------------DVVNQPMKKKAISKLINHCYRTVGLKDTVIFADQLMYTGFAFSTLSGVSIGVNDFVIPTEKARII

*Escherichia* -----------------------------------SIVNQALGKKAISKMLNTCYRILGLKPTVIFADQIMYTGFAYAARSGASVGIDDMVIPEKKHEII

*Mycolicibacterium* -------------------------------------VNEQMHKKVQARIINDLAERFPMIVVAQTVDKLKDAGFYWATRSGVTVSMADVLVPPQKQEIL

*Borrelia* ERANKEIAKIQNDYAKGVITGEERYNNVVSVWLKTNEELTNKMMEILKKDR-----------DGFNVIYMMADSGARGSRNQIRQLAGMRGLMAKTSGDI

*Staphylococcus* DEHEKLVDRITKQFNRGLITEEERYNAVVEIWTDAKDQIQGELMQSLD---------------KTNPIFMMSDSGARGNASNFTQLAGMRGLMAAPSGKI

*Bacillus* EEAQSKVDNVMKQFRRGLITEEERYERVISIWSAAKDVIQGKLMKSLD---------------ELNPIYMMSDSGARGNASNFTQLAGMRGLMANPAGRI

*Clostridioides* AEAEAKVDKYEKAYRRGLISDEERYEKVIETWTETTDKVTDALMGGLD---------------RLNNIYIMAHSGARGSKNQIRQLAGMRGLMANASGKT

*Rhodospirillum* ADTDAEVKEFEQQYQDGLITQGEKYNKVVDAWSHCTERVADEMMKEIAKIE----PG-----KPVNSVYMMAHSGARGSAAQMKQLAGMRGLMAKPSGEI

*Rhodobacter* DAVRDQVKEFEQQYMDGLITQGEKYNKVVDAWSKCSDAVAAEMMKEISAVRV-DDAGAE---KEPNSVYMMSHSGARGSPAQMKQLGGMRGLMAKPSGEI

*Caulobacter* EETRKLAEEYEQQYADGLITKGEKYNKVVDAWAKATDRVADEMMAELQMKHK-DENGRE---KEINAIYMMAHSGARGSQAQMKQLGGMRGLMAKPSGEI

*Pseudomonas* NAATDEVKEIESQYASGLVTQGEKYNKVIDLWSKANDEVSKAMMANLSKEKVVDREGKEVDQESFNSMYMMADSGARGSAAQIRQLAGMRGLMAKPDGSI

*Azotobacter* EGATEEVKEIESQYATGLVTQGEKYNKVIDLWSKANDEVSKAMMANLSKEKVIDRQGNEVEQESFNSMYMMADSGARGSAAQIRQLAGMRGLMAKPDGSI

*Escherichia* SEAEAEVAEIQEQFQSGLVTAGERYNKVIDIWAAANDRVSKAMMDNLQTETVINRDGQEEKQVSFNSIYMMADSGARGSAAQIRQLAGMRGLMAKPDGSI

*Mycolicibacterium* ERHEAEADAIERKYQRGALNHTERNESLVKIWQDATEEVGKALEEFYP---------------ADNPIITIVKSGATGNLTQTRTLAGMKGLVTNPKGEF

*Borrelia* IELPIISNFKEGLSVIEFFISTNGARKGLADTALKTADAGYLTRRLVDIAQDVVVRIEDCGTINGIKVETVKNGEEI--L-ES-LKEKAVGSYSIERIKN

*Staphylococcus* IELPITSSFREGLTVLEYFISTHGARKGLADTALKTADSGYLTRRLVDVAQDVIVREEDCGTDRGLLVSDIKEGTEM--I-EP-FIERIEGRYSKETIHH

*Bacillus* IELPIKSSFREGLTVLEYFISTHGARKGLADTALKTADSGYLTRRLVDVAQDVIIRETDCGTDRGILAKPLKEGTET--I-ER-LEERLIGRFARKQVKH

*Clostridioides* VEIPVKSNFREGLSVLEYFTSSHGARKGLADTAIRTAESGYLTRRLVDVSQDVIVREIDCGTEDTTEIYAIKEGNEV--I-EE-IYDRIVGRYTIDPILN

*Rhodospirillum* IETPIVSNFKEGLTVLEYFNSTHGARKGLADTALKTANSGYLTRRLVDVAQDAIIVIEDCGTSRGITAMPVVEGGQI--I-AS-LGERVLGRTAAEDIKD

*Rhodobacter* IETPIISNFKEGLTVLEYFNSTHGARKGLADTALKTANSGYLTRRLVDVAQDCIVRSHDCGTERAITASAAVNDGEV--V-AP-LSERVLGRVAADDVLV

*Caulobacter* IETPIVSNFKEGLTVQEYFNSTHGARKGLADTALKTANSGYLTRRLVDVAQDCIIVEEDCGTTKGITLRAVVEGGDV--L-VS-LGSRVLGRFTAEDVKD

*Pseudomonas* IETPITANFREGLNVLQYFISTHGARKGLADTALKTANSGYLTRRLVDVAQDLVVTEIDCGTEHGLLMSPHIEGGDV--V-EP-LGERVLGRVIARDVFK

*Azotobacter* IETPITANFREGLNVLQYFISTHGARKGLADTALKTANSGYLTRRLVDVAQDLVVTEIDCGTEQGLLMTPHIEGGDV--V-EP-LGERVLGRVIARDVFR

*Escherichia* IETPITANFREGLNVLQYFISTHGARKGLADTALKTANSGYLTRRLVDVAQDLVVTEDDCGTHEGIMMTPVIEGGDV--K-EP-LRDRVLGRVTAEDVLK

*Mycolicibacterium* IPRPIKSSFREGLTVLEYFINTHGARKGLADTALRTADSGYLTRRLVDVSQDVIVREHDCETERGINVTLAERGPDGTLIRDAHVETSAFARTLATDAVD

*Borrelia* PITGEIVLDANEEISEAKIELLEKIGIEKLVIRSVLTCEAEHGVCQKCYGRDFSKNKPVNIGEAVGIIAAQSIGQPGTQLTMRTFHIGGVAQAGSEDDKI

*Staphylococcus* PETDEIIIRPDELITPEIAKKITDAGIEQMYIRSAFTCNARHGVCEKCYGKNLATGEKVEVGEAVGTIAAQSIGEPGTQLTMRTFHTGGVAG--------

*Bacillus* PETGEVLVNENELIDEDKALEIVEAGIEEVWIRSAFTCNTPHGVCKRCYGRNLATGSDVEVGEAVGIIAAQSIGEPGTQLTMRTFHTGGVAG--------

*Clostridioides* PETGEVIVEADSMIQEDEAETIVALGIEKIRIRTVLNCKTNHGVCSKCYGRNLATGKEVNIGEAVGIIAAQSIGEPGTQLTMRTFHTGGVAG--------

*Rhodospirillum* T-DGTIIVPLGKMIEEHDVELLEEAGIEQVRIRSVLTCEAETGICGKCYGRDLARGTKVNIGEAVGVIAAQSIGEPGTQLTMRTFHIGGAAQRGAEQSSV

*Rhodobacter* PGTDEVLVRKNELIDERKADLIEQNAIQSIRIRSALTCEAEEGVCALCYGRDLARGTLVNQGEAVGIIAAQSIGEPGTQLTMRTFHIGGIAQG-GQQSFL

*Caulobacter* PGTGELVVPADTYIDENIADAIEAAVVQSVKVRSVLTCEAKIGVCGACYGRDLARGTPVNIGEAVGVIAAQSIGEPGTQLTMRTFHIGGTAQV-AEQSFF

*Pseudomonas* PGSDEVIVPAGTLIDEKWVDFLEVMSVDEVVVRSPITCETRHGICAMCYGRDLARGHRVNIGEAVGVIAAQSIGEPGTQLTMRTFHIGGAASRTSAADNV

*Azotobacter* PGGDEVIVPAGTLIDEKWVEFIEMNSIDEVVVRSPITCETRYGICAKCYGRDLARGHQVNIGEAVGVIAAQSIGEPGTQLTMRTFHIGGAASRTSAVDNV

*Escherichia* PGTADILVPRNTLLHEQWCDLLEENSVDAVKVRSVVSCDTDFGVCAHCYGRDLARGHIINKGEAIGVIAAQSIGEPGTQLTMRTFHIGGAASRAAAESSI

*Mycolicibacterium* A-NGNVIIERGHDLGDPAIDALLAAGITTVKVRSVLTCTSATGVCAMCYGRSMATGKLVDIGEAVGIVAAQSIGEPGTQLTMRTFHQGGVTG--------

*Borrelia* SLKNAFILNGIEGFNVRVDNGILFTRKGTLKIINVFYEEKIKNIKEIKVLDSQRVIKGIPLFIDKKGSEILSSYIGYVKLRDDNFFIVSEEQEVSLKAGT

*Staphylococcus* ----------------------------------------------------------------------------------------------------

*Bacillus* ----------------------------------------------------------------------------------------------------

*Clostridioides* ----------------------------------------------------------------------------------------------------

*Rhodospirillum* EAAF----------------------DGKIVMENR------------------------AVVGTSENVLIVMSRNCEVKITDEA---GREKARYRIPYGS

*Rhodobacter* EASQ----------------------EGKIEFRNA------------------------NLLINDAGEQIVMGRNMQLAIVSEQ---GEERASHKLSYGS

*Caulobacter* EASN----------------------EGTVRVIGP------------------------T-VVGSDGALVIMSRNTSVSVLVD----GKERETYKPPYGA

*Pseudomonas* QVKN----------------------GGTIRLHNL------------------------KHVVRADGALVAVSRSGELAVADDF---GRERERYKLPYGA

*Azotobacter* QVKN----------------------GGNIRLHNL------------------------KYVERSDGALVAVSRSGELAVADDF---GRERERYKLPYGA

*Escherichia* QVKN----------------------KGSIKLSNV------------------------KSVVNSSGKLVITSRNTELKLIDEF---GRTKESYKVPYGA

*Mycolicibacterium* ----------------------------------------------------------------------------------------------------

*Borrelia* KLEIEVGDYVESGKVIGTFDPFAEPIIAEVKGKIKFKDIILGTTLKEEINTETGNVEKRITDNV-----FESLDPRIFIIDSSGMEV--------ASYVL

*Staphylococcus* ----------------------------------------------------------------------------------------------------

*Bacillus* ----------------------------------------------------------------------------------------------------

*Clostridioides* ----------------------------------------------------------------------------------------------------

*Rhodospirillum* KLLTDEGRMVTKGDRLAEWDPYTVPIITEREGIAIYNDLVEGVSVREVTDEATGISSKVVVEWKNM-PKGTDLKPRITLRDDRGEGITLA-NGLEARYFM

*Rhodobacter* KIFVKDGDAVKRGTKLFEWDPYTLPIIAEKAGVAKFVDLISGIAVREETDEATGMTQKIVTDWRSA-PKGNDLKPEIIIVNADGEPVRND-AGNPVTYPM

*Caulobacter* RLRVKDGDLVKRGQRLGDWDPYTTPIITEVAGKIRAEDLVDGLSIREEVDEATGIAQRVVADWRTS-ARGSDLRPAMGVLSEDGSYKRLS-NGGEARYLL

*Pseudomonas* VISVKEGDKVDPGAIVAKWDPHTHPIVTEVDGTVAFVGMEEGITVKRQTDELTGLTNIEVMDPKDRPAAGKDIRPAVKLIDAAGKDLLLPGTDVPAQYFL

*Azotobacter* VISVKEGDKVSPGAIVAKWDPHTHPIVTEMKGTVTFVGMEEGITIKRQTDELTGLTNIEVIDPKDRPAAGKDIRPAVKLVDASGKELLLPGTDVPAQYFL

*Escherichia* VLAKGDGEQVAGGETVANWDPHTMPVITEVSGFVRFTDMIDGQTITRQTDELTGLSSLVVLDSAERTAGGKDLRPALKIVDAQGNDVLIPGTDMPAQYFL

*Mycolicibacterium* ----------------------------------------------------------------------------------------------------

*Borrelia* PGDAYLQVEDGQSINIGDIIAKLSKGSEKTQDITGGLPRVNDLFETRIPKNLTEMAKVSGIVQFKSIQKGKRLINILDE---YG---VEH-KHYIPAGKH

*Staphylococcus* ------------------------------SDITQGLPRIQEIFEARNPKGQAVITEIEGVVEDIKLAKDRQQEIVVKG---AN---ETR-SYLASGTSR

*Bacillus* ------------------------------DDITQGLPRIQELFEARNPKGQATITEIDGTVVEINEVRDKQQEIVVQG---AV---ETR-SYTAPYNSR

*Clostridioides* ------------------------------ADITQGLPRVEELFEARKPKGLAVITEVSGRVEI-DETGKRKEVNVIPE---EG---ETQ-TYVIPYGSR

*Rhodospirillum* SVDTILSVENGQRVKAGDVLGRIPREGSKTRDITGGLPRVAELFEARKPKDHAIISEIDGRVEFGKDYKSKRRLLVVPE---DG---DPV-EYLLPKGKH

*Rhodobacter* SVDAILSIEDGQDVKAGDVVARIPREGAKTKDITGGLPRVAELFEARRPKDHAIIAELDGYVRFGKDYKNKRRITIEPSQ--DGL--EPV-EYMVPKGKH

*Caulobacter* SAGAILSVADGDEVKPGEVIARIPTEGAKTRDITGGLPRVAELFEARRPKDCAVIAEMDGRVEFGKDYKNKRRIKITPDVDADGNQPEAV-EFLIPKGKH

*Pseudomonas* PANALVNLTDGAKVSIGDVVARIPQETSKTRDITGGLPRVADLFEARRPKEPSILAEISGTISFGKETKGKRRLVITPN---DGS--DPY-EELIPKWRH

*Azotobacter* PANALVGVADGAEVGVGDVIARIPQETSKTRDITGGLPRVADLFEARRPKEPSILAEVSGTISFGKETKGKRRLVITPN---DGS--EAY-EELIPKWRH

*Escherichia* PGKAIVQLEDGVQISSGDTLARIPQESGGTKDITGGLPRVADLFEARRPKEPAILAEISGIVSFGKETKGKRRLVITPV---DGS--DPY-EEMIPKWRQ

*Mycolicibacterium* -----------------------------GADIVGGLPRVQELFEARVPRNKAPIADVAGRVRLEESDKF-FKITIVPD---DGG--EEVVYDKLSKRQR

*Borrelia* L-----------LVRDGDVVKAGDMLCDGRINPHDVLEILGGISLQEFLLAEIQDVYRKQGVSINDKHIGVIIKQMMKKVKIVAVGDTNFVYGQKVDKHT

*Staphylococcus* I-----------IVEIGQPVQRGEVLTEGSIEPKNYLSVAGLNATESYLLKEVQKVYRMQGVEIDDKHVEVMVRQMLRKVRIIEAGDTKLLPGSLVDIHN

*Bacillus* L-----------KVAEGDKITRGQVLTEGSIDPKELLKVTDLTTVQEYLLHEVQKVYRMQGVEIGDKHVEVMVRQMLRKVRVIDAGDTDVLPGTLLDIHQ

*Clostridioides* L-----------KVKQGQMLEAGDPLTQGFINPHDIVRVNGVKGVQEYIVKEVQRVYRLQGVDVNDKHIEVIVRQMLSKVKVEDPGDTDLLPGGYEDVLT

*Rhodospirillum* L-----------TIQEGDYVRKGDPLMDGNPVPHDILRVMGVEALANYLIKEIQDVYRLQGVKINDKHIEVISRQMLQKVEITEPGDTTFLVGELIDRTD

*Rhodobacter* I-----------PVQEGDFVQKGDYIMDGNPAPHDILRIMGIEALAEYLIDEVQDVYRLQGVKINDKHIEVIVRQMLQKIEILDSGDTTLLKGEHIDKSE

*Caulobacter* I-----------AVHDGDYITKGEYIIDGNPDPHDILRILGVEALANFLVDEIQEVYRLQGVPINDKHIETIVRQMLQKVEILEPGDTGLIKGDHLDKPE

*Pseudomonas* L-----------NVFEGEQVNRGEVISDGPSNPHDILRLLGVSSLAKYIVNEIQDVYRLQGVKINDKHIETILRQMLRKVEVSESGDSSFIKGDQVELTQ

*Azotobacter* L-----------NVFEGEQVNRGEVISDGPSNPHDILRLLGVSALAKYIVNEIQDVYRLQGVKINDKHIETILRQMLRKVEITEAGDSSFIKGDQVELTQ

*Escherichia* L-----------NVFEGERVERGDVISDGPEAPHDILRLRGVHAVTRYIVNEVQDVYRLQGVKINDKHIEVIVRQMLRKATIVNAGSSDFLEGEQVEYSR

*Mycolicibacterium* LRVITHEDGTEGVLSDGDHVEVGDQLMEGAADPHEVLRVQGPREVQIHLVKEVQEVYRAQGVSIHDKHIEVIVRQMLRRVTIIDSGSTEFLPGSLTERAE

*Borrelia* FYEQNRKVIEQGGEPAIASPILIGVTKTSLNIDSFISAASFQETTKVLTDASIAGKIDDLRGLKENVVIGHLIPTGTGMGLYKKIKVSENID--------

*Staphylococcus* FTDANREAFKHRKRPATAKPVLLGITKASLETESFLSAASFQETTRVLTDAAIKGKRDDLLGLKENVIIGKLIPAGTGMRRYSDVKYEKTAK--PV-A--

*Bacillus* FTEANKKVLLEGNRPATGRPVLLGITKASLETDSFLSAASFQETTRVLTDAAIKGKRDELLGLKENVIIGKLVPAGTGMMKYRKVKPVSNVQ--PT-D--

*Clostridioides* FNECNKDAIDKGLRPAVAKRVLLGITKASLATDSFLSAASFQETTRVLTEAAIKGKEDHLIGLKENVILGKLIPAGTGMKKYRNIAVEKI----------

*Rhodospirillum* FQIENEKTLKENGRPANSIPVLQGITKASLQTHSFISAASFQETTRVLTEAAVSGKSDSLMGLKENVIVGRLIPAGTGAMMNRLRALAATRDK-EI-E--

*Rhodobacter* FDEENAKVEARGGRAATGEPVLLGITKASLQTRSFISAASFQETTRVLTEASVQGKRDKLVGLKENVIVGRLIPAGTGGATTRVRKIAAERDS-KV-I--

*Caulobacter* FDKEQEKAIARGGRPAVTQPVLLGITKASLQTKSFISAASFQETTRVLTEASVHGKTDTLEGLKENVIVGRLIPAGTGSYLRSLQRVAAKRDE-QL-A--

*Pseudomonas* VLEENEQLGTEDKFPAKYERVLLGITKASLSTESFISAASFQETTRVLTEAAVTGKRDFLRGLKENVVVGRLIPAGTGLAYHSERKRQRDLGK-P--Q--

*Azotobacter* VLEENEQLATEDRFSAKYERILLGITKASLSTESFISAASFQETTRVLTEAAVTGKRDYLRGLKENVVVGRLIPAGTGLAYHSERRRKRDAEK-P--V--

*Escherichia* VKIANRELEANGKVGATYSRDLLGITKASLATESFISAASFQETTRVLTEAAVAGKRDELRGLKENVIVGRLIPAGTGYAYHQDRMRRRAAGEAPAAP--

*Mycolicibacterium* FEAENRRVVAEGGEPAAGRPVLMGITKASLATDSWLSAASFQETTRVLTDAAINCRSDKLNGLKENVIIGKLIPAGTGISRYRNIQVQPTEEA-RAAAYT

*Borrelia* --------------------------------------------------SEV

*Staphylococcus* ------------EVE----SQ-------------T--------------EVTE

*Bacillus* ------------D---------------------M--------------VPVE

*Clostridioides* ---------------------------------------------------ED

*Rhodospirillum* ------------DSRGAEMVPVLGAAESFTPRLPE--------------PPAE

*Rhodobacter* ------------EARRAEAIDAAALAA--PIDTAY---GDDDFGPVETPESRD

*Caulobacter* ------------QQRE-DAMEPL------PAEIAL--------------SDAE

*Pseudomonas* ------------RVSA-SEAEA-ALTE-----ALN--------------SSGN

*Azotobacter* ------------RVSA-EEVEA-ALTE-----ALN--------------TSGN

*Escherichia* ------------QVTA-EDASA-SLAE-----LLNAGLGG---------SDNE

*Mycolicibacterium* IPSYEDQYYSPDFGQA-TGAAV-PLDD-----YGY--------------SDYR

**Original, uncropped Western blots and phosphor screen gels.** Red squares indicate cropped section selected for the final figures. Gels without cropping were used for quantitative analysis displayed in the original figure.


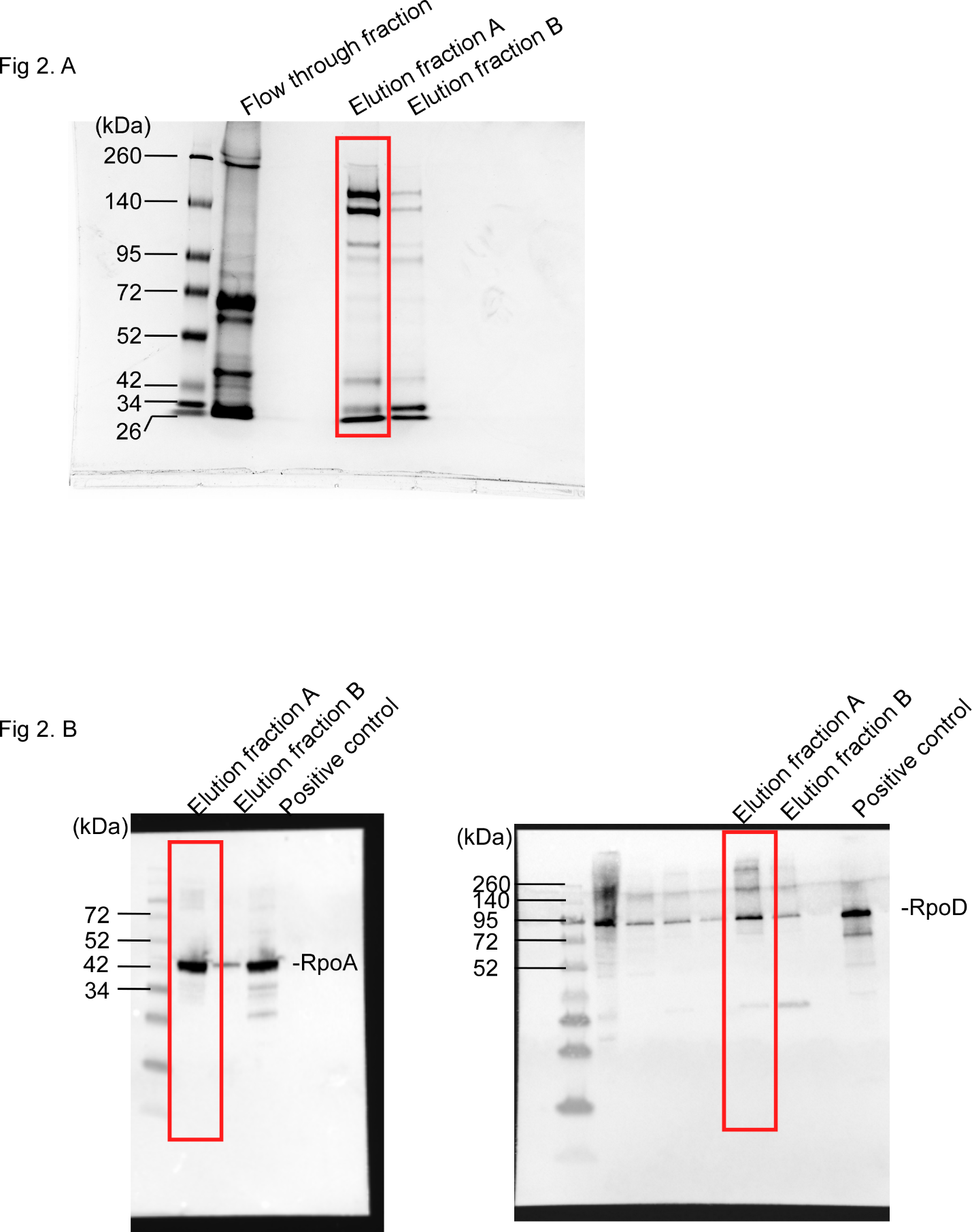


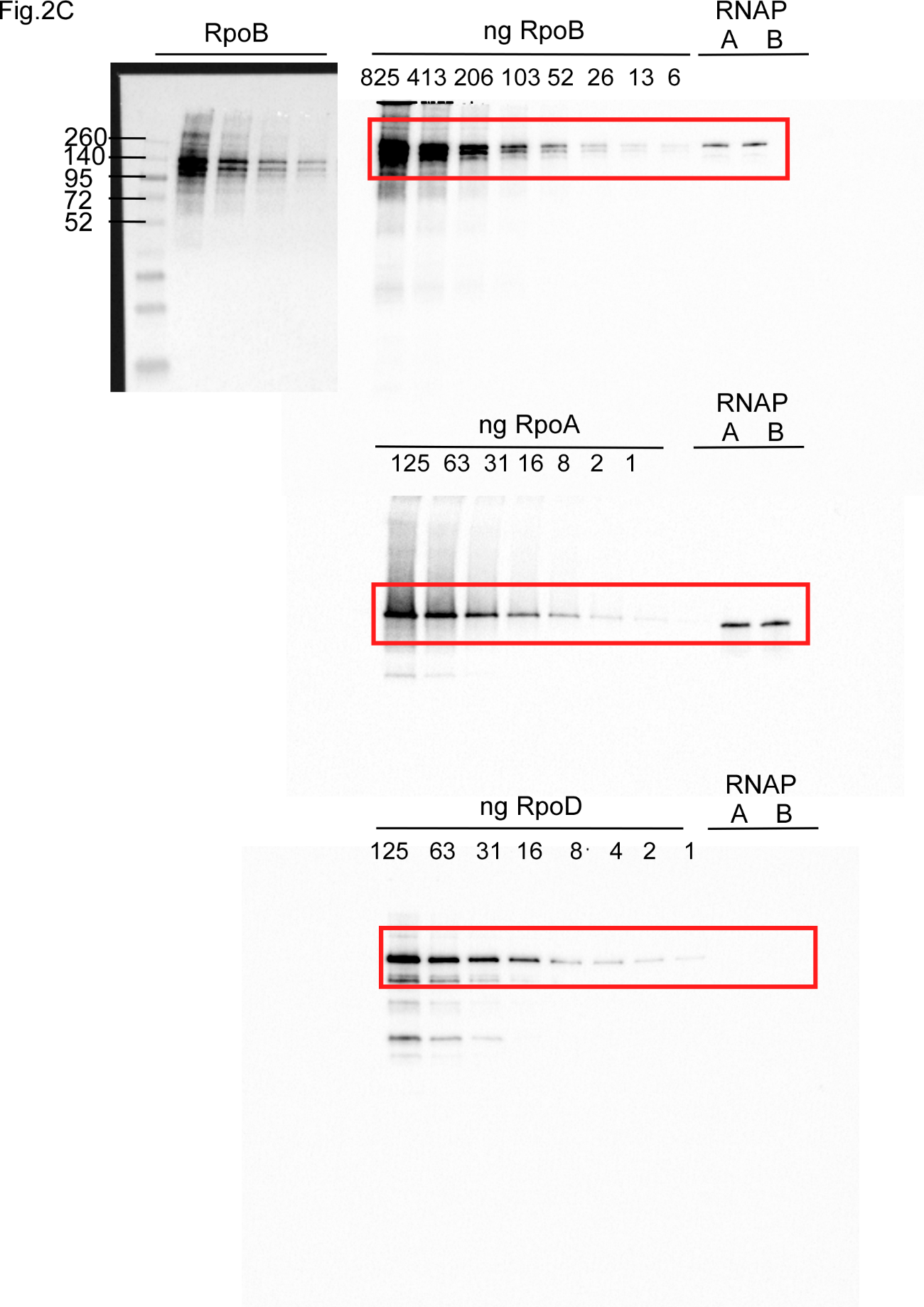

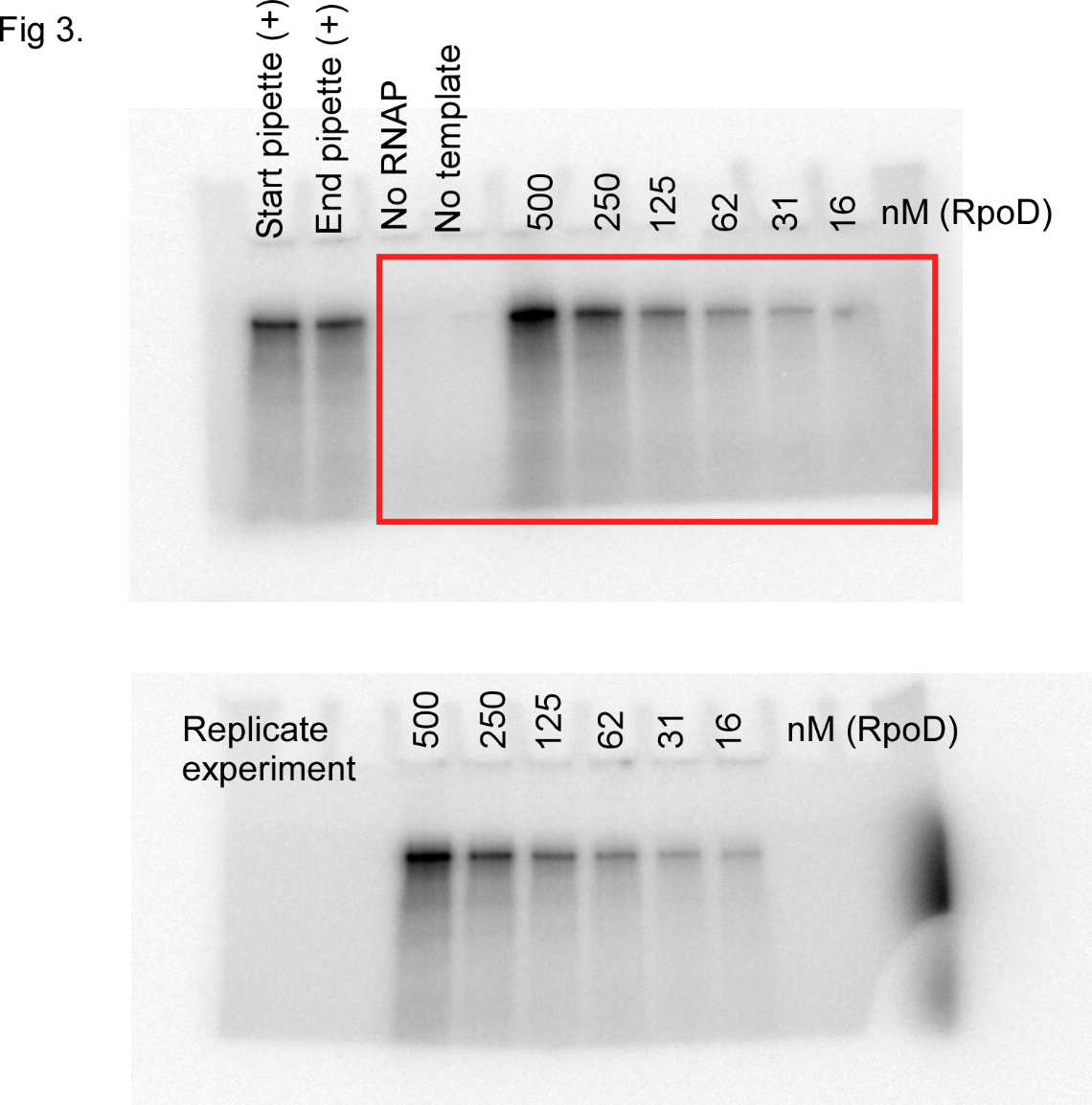


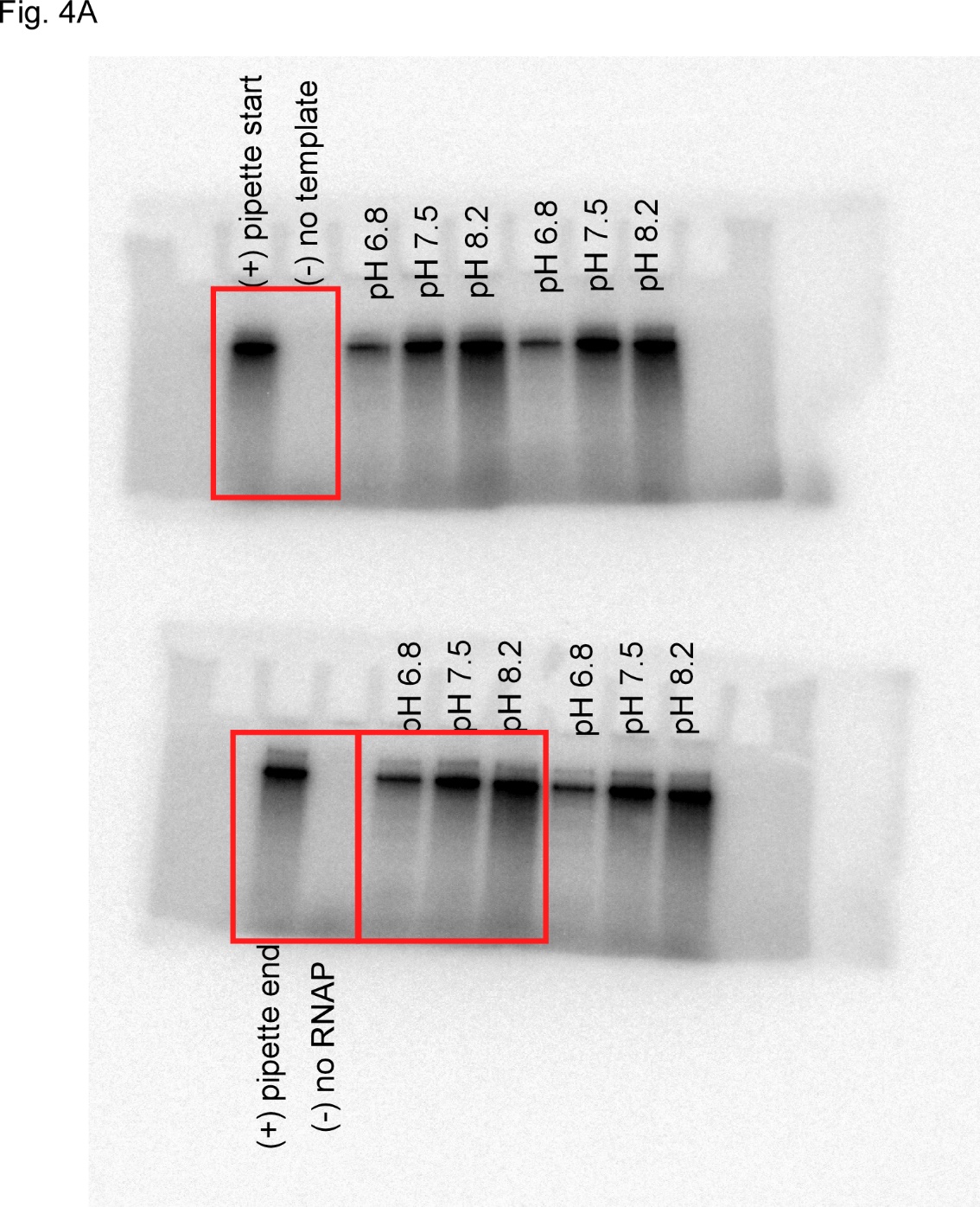


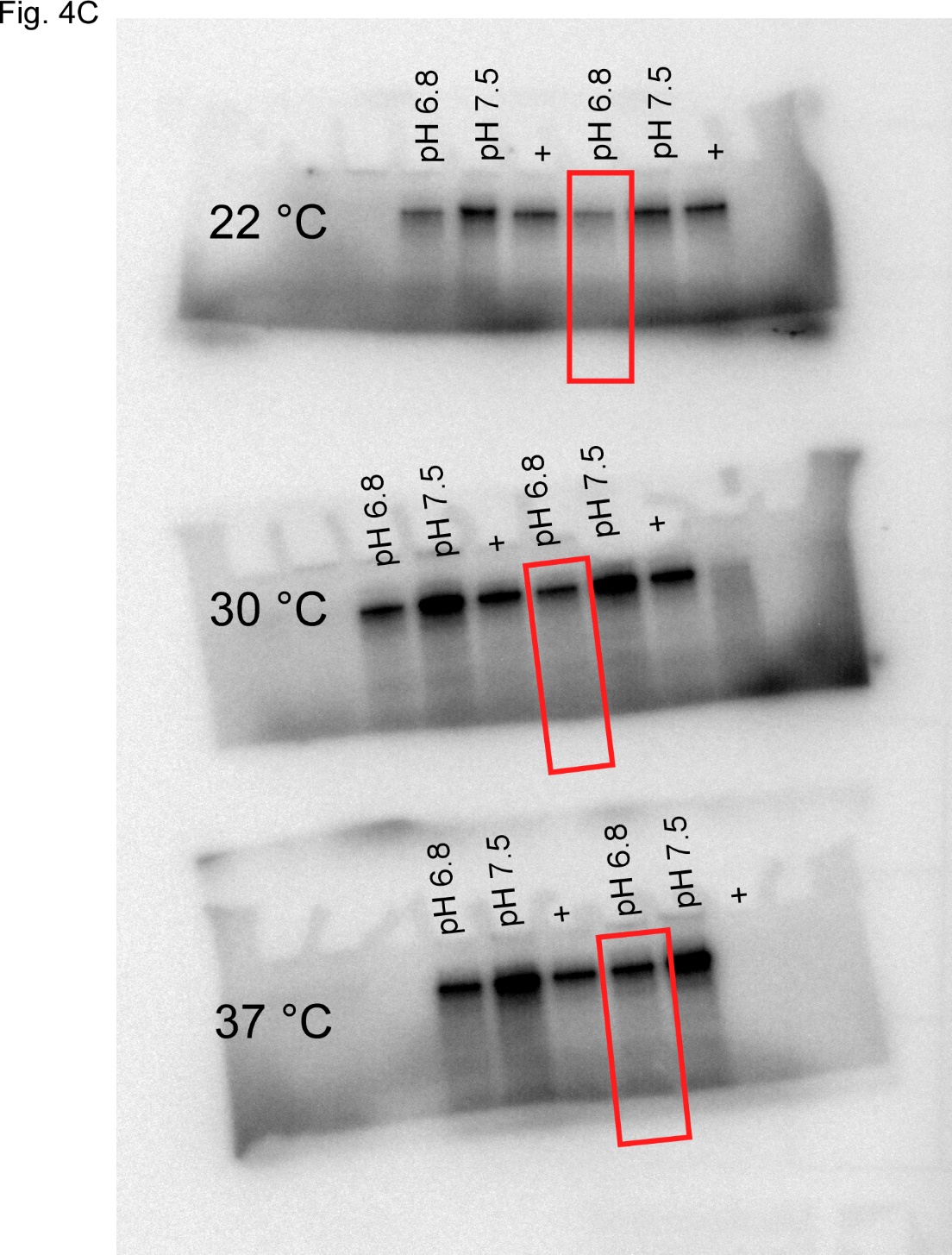


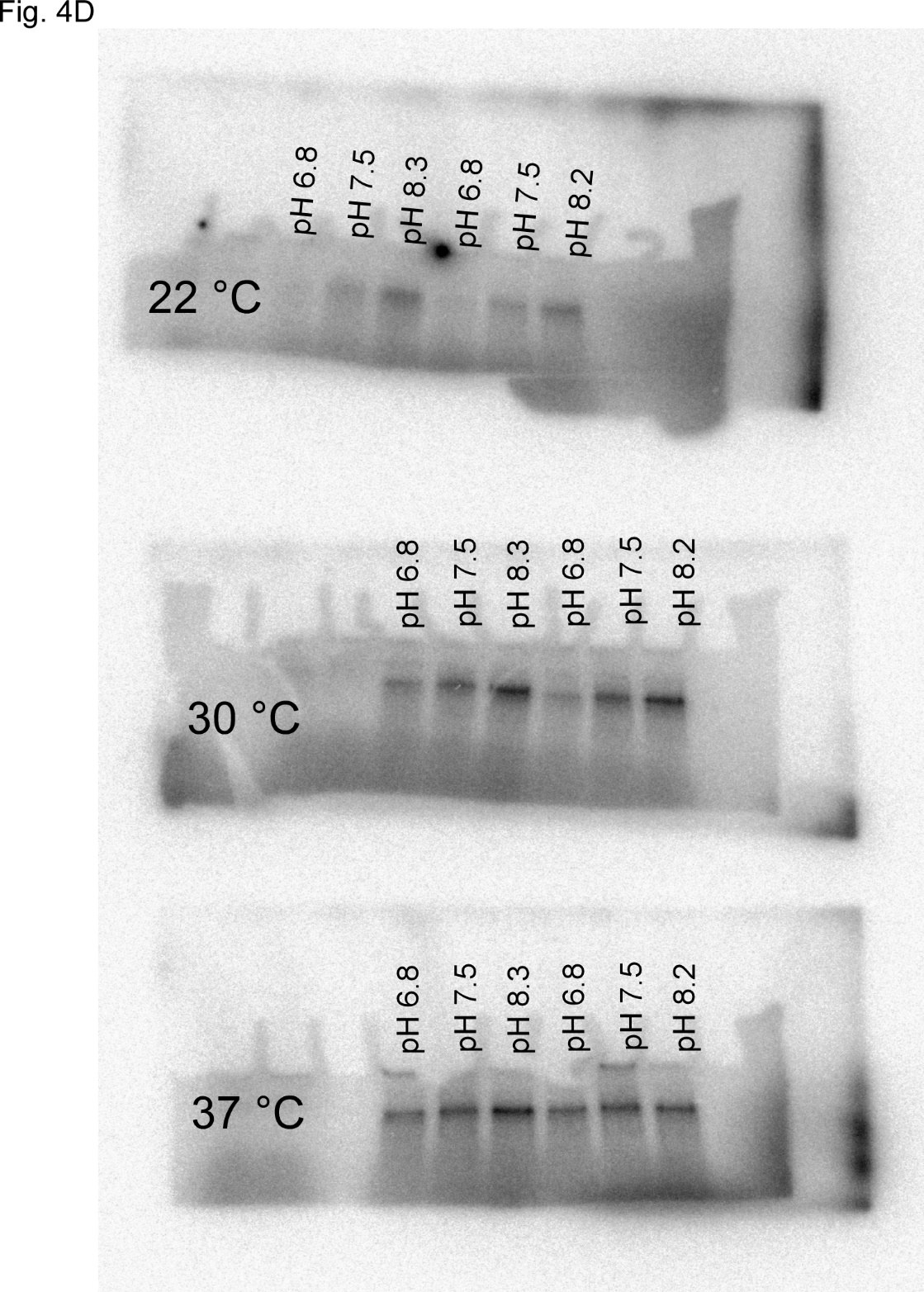

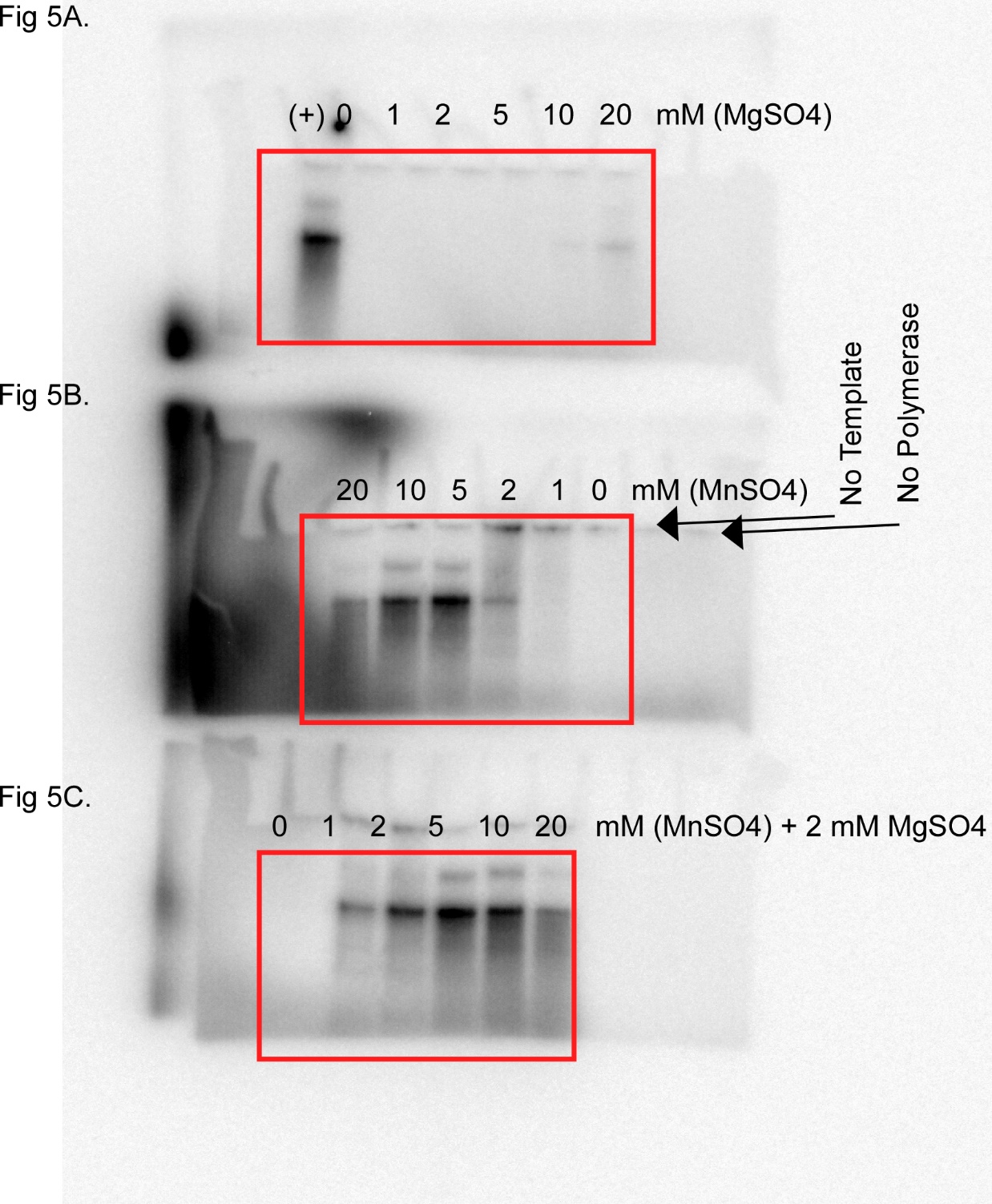


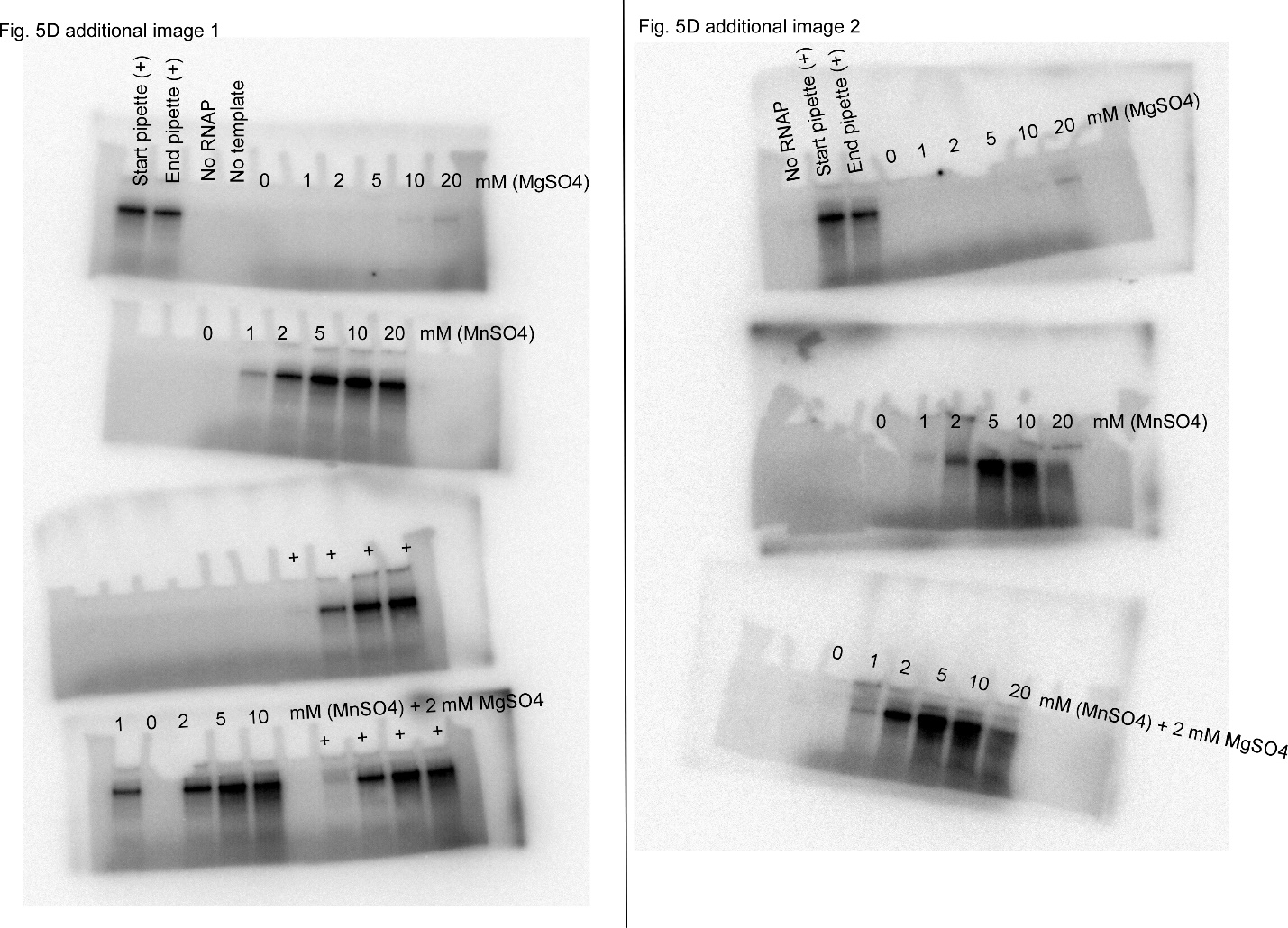

Supplement: Supplementary file 1 — Supplementary information. [file 41598_2020_65104_MOESM1_ESM.docx]
